# Dynamics and Mechanisms in the Recruitment and Transference of Histone Chaperone CIA/ASF1

**DOI:** 10.3390/ijms20133325

**Published:** 2019-07-06

**Authors:** Yanjun Zhang, Huanyu Tao, Sheng-You Huang

**Affiliations:** School of Physics, Huazhong University of Science and Technology, Wuhan 430074, China

**Keywords:** protein–protein interactions, molecular dynamics, dynamic pathway, histone chaperone

## Abstract

The recruitment and transference of proteins through protein–protein interactions is a general process involved in various biological functions in cells. Despite the importance of this general process, the dynamic mechanism of how proteins are recruited and transferred from one interacting partner to another remains unclear. In this study, we investigated the dynamic mechanisms of recruitment and translocation of histone chaperone CIA/ASF1 for nucleosome disassembly by exploring the conformational space and the free energy profile of unbound DBD(CCG1) and CIA/ASF1-bound DBD(CCG1) systems through extensive molecular dynamics simulations. It was found that there exists three metastable conformational states for DBD(CCG1), an unbound closed state, a CIA/ASF1-bound half-open state, and an open state. The free energy landscape shows that the closed state and the half-open state are separated by a high free energy barrier, while the half-open state and the open state are connected with a moderate free energy increase. The high free energy barrier between the closed and half-open states explains why DBD(CCG1) can recruit CIA/ASF1 and remain in the binding state during the transportation. In addition, the asymmetric binding of CIA/ASF1 on DBD(CCG1) allows DBD(CCG1) to adopt the open state by moving one of its two domains, such that the exposed domain of DBD(CCG1) is able to recognize the acetylated histone H4 tails. As such, CIA/ASF1 has a chance to translocate from DBD(CCG1) to histone, which is also facilitated by the moderate energy increase from the bound half-open state to the open state of DBD(CCG1). These findings suggest that the recruitment and transference of histone chaperone CIA/ASF1 is highly favored by its interaction with DBD(CCG1) via conformational selection and asymmetric binding, which may represent a general mechanism of similar biological processes.

## 1. Introduction

Protein–protein interactions play an important role in many biological processes [1,2,3,4,5,6,7,8], in which the recruitment and transference is a general process to conduct various biological functions in cells. Despite the importance of this general process, the dynamic mechanism of how proteins are recruited and transferred from one interacting partner to another remains unclear. For example, the nucleosome—the universal repeating unit of chromatin—mainly contains 147 base pairs of DNA wrapped around an octamer of two copies each of histones H2A, H2B, H3, and H4 [9,10,11,12,13,14,15,16]. The tightly orchestrated assembly (deposition of histone proteins onto naked DNA) or disassembly (removal of histone proteins from DNA) process is the most severe alteration of chromatin structure, which is mainly mediated by histone chaperone proteins in the nucleus [17,18,19,20,21,22,23,24,25,26,27,28,29]. CIA/ASF1 [cell cycle gene 1 (CCG1)-interacting factor A or antisilencing function 1] is a highly conserved histone chaperone implicated in nucleosome assembly and disassembly, transcriptional silencing, and the cellular response to DNA damage [17,21,30,31,32,33,34,35,36,37]. The dysfunction of CIA/ASF1 would cause severe replication defects and loss of chromatin integrity [38,39]. To achieve its biological function, CIA/ASF1 needs to interact with many other specific macromolecular sites [21,33,40,41]. The site-specific histone eviction from the nucleosome by CIA/ASF1 at the transcription initiation process is generally induced by the histone acetylation around the active promoter region.

**Figure 1:**
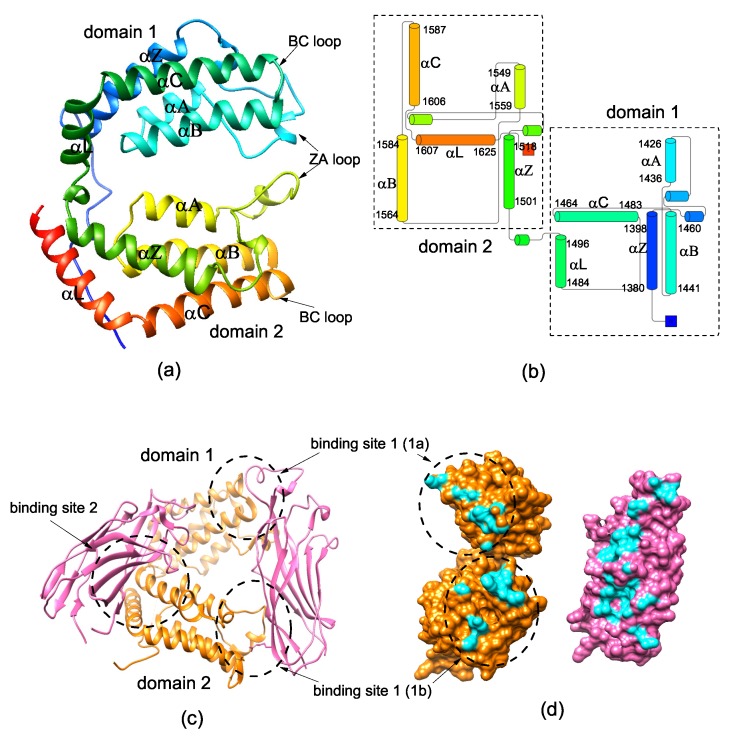
Crystal structure of unbound DBD(CCG1) and CIA/ASF1-bound DBD(CCG1). (**a**) Ribbon representation of unbound DBD (PDB ID: 1EQF). (**b**) Topology and delimiting sequence markers of unbound DBD(CCG1). (**c**) Ribbon representation of CIA/ASF1-bound DBD(CCG1). (**d**) Binding surface of DBD(CCG1) and CIA/ASF1. DBD(CCG1) is colored in orange, CIA/ASF1 is colored in pink, and the binding interface is colored in cyan.

It has been revealed that double bromodomain in the CCG1/TAF1/TAF(II)250 subunit [DBD(CCG1)] of the general transcription initiation factor TFIID, which has the ability to recognize the acetylated histone *N*-terminal region of histone H4, plays a critical role in recruiting CIA/ASF1 to the promoter regions [34,42,43,44,45,46]. The binding of DBD(CCG1) to CIA/ASF1 is essential for nucleosome assembly and disassembly. DBD(CCG1) consists of two tandem bromodomain modules and the core of each bromodomain is a four-helix bundle (Figure 1a,b) [43]. The experimental structure of the CIA/ASF1-DBD(CCG1) complex indicates that DBD(CCG1) has two binding sites to interact with CIA/ASF1 [42] (Figure 1c). The interaction between CIA/ASF1 and binding site 1 of DBD(CCG1) is essential for their colocalization at active promoter sites. There exists a large conformational change between the unbound (PDB ID: 1EQF) and CIA/ASF1-bound (PDB ID: 3AAD) structures of DBD(CCG1). It is shown that the conformational change is mainly caused by the relative movement of two bromodomains, which is caused by the binding of CIA/ASF1 at binding site 1. The binding site 1 of DBD(CCG1) is a hydrophobic pocket formed by the conserved residues of ZA loops and BC loops [42,43]. The acetylated lysine-binding site of DBD(CCG1) is also located at the hydrophobic pocket, but the acetylated lysine-binding residues are mostly exposed to the solvent, which indicates that the binding of CIA/ASF1 does not prevent DBD(CCG1) from interacting with the acetylated lysine [47]. When DBD(CCG1) brings CIA/ASF1 to the promoter regions through the interaction between DBD(CCG1) and the acetylated *N*-terminal tail of histone H4, CIA/ASF1 would change its interacting partner from DBD(CCG1) to histone H3-H4 [17,48,49]. The crystal structure shows that the hydrophobic interaction regions (the site-edge pocket and the concave surface of a β-sheet) of CIA/ASF1 with histone H3-H4 and DBD(CCG1) are overlapped [42,50] (Figure 1d). Yusuke et al. suggested that the histone H3-H4 has a larger interaction surface with CIA/ASF1 than DBD(CCG1), which leads to the formation of CIA/ASF1-H3-H4 complex [42]. However, the dynamic pathway of the transference, i.e., how CIA/ASF1 changes its interacting partner from DBD(CCG1) to histone H3-H4, remains unclear.

Herein, we have performed extensive molecular dynamics (MD) simulations on unbound and CIA/ASF1-bound DBD(CCG1) structures to gain insights into the dynamics process of the recruitment and transference of CIA/ASF1. It was found that there exists three metastable conformational states for DBD(CCG1)—the unbound closed state, the CIA/ASF1-bound half-open state, and the open state. The dynamic mechanism of the recruitment and transference of CIA/ASF1 was extensively investigated through the conformational states of bound and unbound DBD(CCG1) and their free energy profiles.

## 2. Results

### 2.1. There Exists Three Metastable Conformational States for DBD(CCG1)

**Figure 2:**
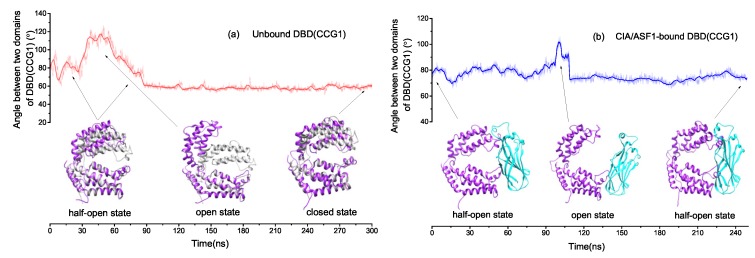
The angle curves between domain 1 and domain 2 of DBD(CCG1) for unbound DBD(CCG1) and CIA/ASF1-bound DBD(CCG1) versus time. The structures extracted from MD simulations are colored in purple, the crystal structure of unbound DBD(CCG1) is colored in gray, and the CIA/ASF1 is colored in cyan.

The previous studies revealed that DBD(CCG1) shows distinct conformations between free and CIA/ASF1-bound DBD(CCG1) structures [42]. The angle between the principal axes of domains 1 and 2 of DBD(CCG1) increases about 10${}^{\circ }$ upon CIA/ASF1 binding. In order to get insights into the conformational dynamics of DBD(CCG1), we calculated the angle between domain 1 and domain 2 versus time (Figure 2) and the root mean square deviation (RMSD) of DBD(CCG1) relative to its initial crystal structure (Figure S1), where the RMSD is calculated based on their C${}_{\alpha }$ atoms. It is shown that the unbound DBD(CCG1) appears to exhibit three metastable conformational states according to the angle curve, which we denote as the unbound closed state, the CIA/ASF1-bound half-open state, and the open state (Figure 2a). Comparing with the initial crystal structure, the angle between the principal axes of domains 1 and 2 and the RMSD of the open state increase about 30${}^{\circ }$ and 5 Å, though the domains 1 and 2 themselves do not show significant conformational change. The unbound closed state is very close to the experimental structure of free DBD(CCG1) (PDB ID: 1EQF). Figure 2b shows that the angle between domain 1 and domain 2 of CIA/ASF1-bound DBD(CCG1) has a very small fluctuation, except at 95–110 ns, where the angle shows a large increase and then drops to about the previous level. We also extracted one frame from the simulations for 95–110 ns. It shows that DBD(CCG1) around this time exhibits a large conformational change, which is very similar to the open state of unbound DBD(CCG1). The RMSDs for unbound and CIA/ASF1-bound DBD(CCG1) confirm the binding state change of DBD(CCG1) (Figure S1a,b). The conformational change from the half-open state to the open state enables the binding site 1a of DBD(CCG1) to expose to solvent, which gives DBD(CCG1) opportunities to recognize the acetylated *N*-terminal tail of histone H4 at this site.

**Figure 3:**
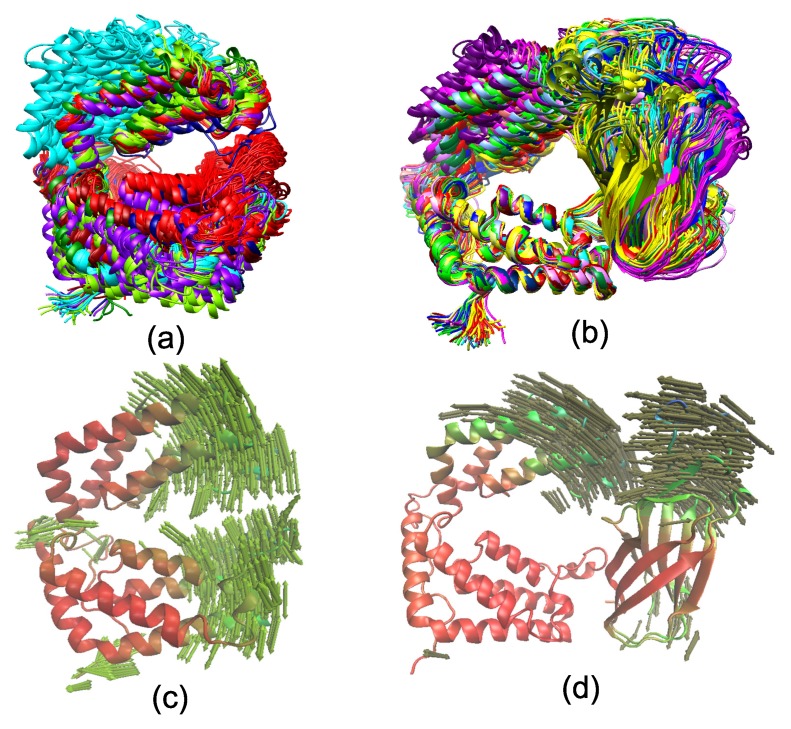
Ensemble cluster analysis of (**a**) unbound DBD(CCG1) and (**b**) CIA/ASF1-bound DBD(CCG1). Principal component analysis of (**c**) unbound DBD(CCG1) and (**d**) CIA/ASF1-bound DBD(CCG1). CIA/ASF1-bound DBD(CCG1) has two binding states, half-open state and open state, and the binding state changes are mainly caused by the movement of domain 1.

The ensemble cluster analysis was also used to study the conformational transition of DBD(CCG1) in unbound and CIA/ASF1-bound DBD(CCG1), by using the frames from the whole trajectories [51]. The corresponding results are shown to be consistent with the results of angles between two domains and RMSDs. For unbound DBD(CCG1), domains 1 and 2 can switch among the three binding states, and about 60% of the snapshots belong to the closed state, which suggests that the closed state is relatively stable for unbound DBD(CCG1) (Figure 3a). The CIA/ASF1-bound DBD(CCG1) system only exhibits two metastable conformational states, the half-open state and the open state, while the closed state does not present (Figure 3b). When the system changes from the half-open to the open state, domain 1 of DBD(CCG1) has a significant conformational translation. Meanwhile, the CIA/ASF1 also rotates about 10${}^{\circ }$ around binding site 1b, such that the exposed site of CIA/ASF1 would be able to bind to histone H3-H4. The results of PCA reveal that the transition among different conformational states of unbound DBD(CCG1) is mainly due to the relative movement of domain 1 and domain 2 (Figure 3c). Unlike unbound DBD(CCG1), the open state of CIA/ASF1-bound DDB(CCG1) is mostly caused by the movement of domain 1 and the rotation of CIA/ASF1 (Figure 3d). In the whole process, CIA/ASF1 tightly binds to binding site 1b that serves as a hinge for the conformation of DBD(CCG1) to change from one conformational state to the other. As observed in the crystal structure of CIA/ASF1-bound DBD(CCG1), binding site 1b has a larger binding interface than binding site 1a. The interface between binding site 1b and CIA/ASF1 also involves more hydrophobic interactions than that between binding site 1a and CIA/ASF1 (Table S1). From the binding free energy analysis, it can also be found that the energy contributions from binding site 1b (−31.27 kcal/mol) are 3.6 times more than that from binding site 1a (−8.69 kcal/mol). The hydrogen bonds are mainly formed between binding site 1b and CIA/ASF1 (Table S3). These results suggest a much more stable binding of domain 2 than domain 1 of DBD(CCG1) to CIA/ASF1, which will also be discussed in Section 2.3.

The finding that the unbound DBD(CCG1) has three metastable conformational states, the unbound closed state, the CIA/ASF1-bound half-open state, and the open state, is consistent with the experimental results. The open state of CIA/ASF1-bound DBD(CCG1) is a new binding state, which was not reported before. This open state is expected to play a key role in the transference of CIA/ASF1. That is, because the interacting regions of CIA/ASF1 with DBD(CCG1) and H3-H4 are overlapped, the open state would give a chance for DBD(CCG1) to recognize the acetylated *N*-terminal tail of histone H4 and then for CIA/ASF1 to change its interacting partner from DBD(CCG1) to histone H3-H4. This reveals a possible mechanism in the transference of CIA/ASF1 from DBD(CCG1) to histone H3-H4 and may help us further understand the process of nucleosome assembly and disassembly.

### 2.2. Exploring the Free Energy Landscape

From the previous studies [42,43,46] and our present MD simulations, it has been shown that DBD(CCG1) may transit among three distinct metastable conformational states—the closed state, the half-open state, and the open state. The unbound DBD(CCG1) mostly stays in the closed state. Induced by the binding of CIA/ASF1 [42], DBD(CCG1) will experience a large conformational change, such that the angle between the principal axes of domains 1 and 2 increases about 10${}^{\circ }$ compared with the closed state, resulting in a bound half-open state. The open state of CIA/ASF1-bound DBD(CCG1) is a new state revealed through our MD simulations, which has never been reported by experiment. The open state of DBD(CCG1) is crucial for the transference of CIA/ASF1 as it gives CIA/ASF1 the chance to change its interacting partner from DBD(CCG1) to histone H3-H4. To further investigate the molecular mechanism of CIA/ASF1 interacting with DBD(CCG1) during the recruitment and transference, we have explored the free energy landscape of the system.

**Figure 4:**
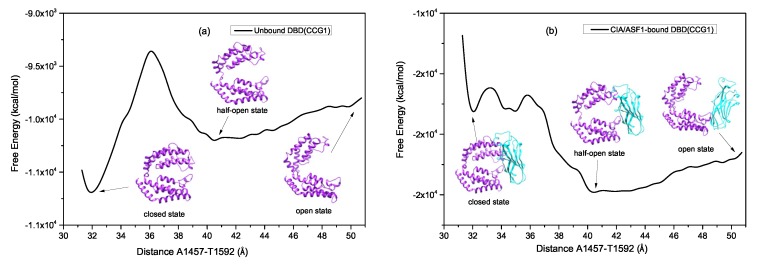
Free energy landscape in the conformational coordinate (the distance between A1457 C${}_{\alpha }$ and T1592 C${}_{\alpha }$). (**a**) Unbound DBD(CCG1). (**b**) CIA/ASF1-bound DBD(CCG1) complex.

To obtain the free energy profile, we first constructed the models of unbound DBD(CCG1) and CIA/ASF1-bound DBD(CCG1) from the closed state to the open state. The bound half-open state was based on the crystal structure of PDB ID: 3AAD [42], and the closed state was based on that of PDB ID: 1EQF [43]. Comparing the crystal structures of CIA/ASF1-bound and unbound DBD(CCG1) shows that DBD(CCG1) undergoes a global conformational change upon CIA/ASF1 binding, where the binding state changes from the closed state to the half-open state, although the closed state of CIA/SF1-bound DBD(CCG1) wild type (WT) is not easily accessible in MD simulations due to its high free energy. Therefore, the unbound closed state of CIA/ASF1-DBD(CCG1) was modeled based on the unbound crystal structure of DBD(CCG1). The open state was based on the open conformation extracted from the MD trajectory. The pathway of conformational transition was based on our MD simulations. Forty models were constructed from the unbound closed state to the open state to calculate the free energy landscape. The total free energy of the models were calculated by the Molecular Mechanics Generalized Born Surface Area (MM-GBSA) method. Figure 4 shows the one-dimensional (1D) free energy landscape from the unbound closed state to the open state of the unbound and CIA/ASF1-bound DBD(CCG1) systems. As indeed shown in the free energy profile, there are three local energy minima corresponding to the three metastable conformational states, where there is a high energy barrier between the closed state and the half-open state and only a moderate energy increase from the half-open state to the open state (Figure 4).

#### 2.2.1. The High Energy Barrier between the Closed and Half-Open Bound States of DBD(CCG1) Enables CIA/ASF1 to Stably Bind to the Half-Open State during the Recruitment

As shown in the free energy landscape of unbound DBD(CCG1), we find that the closed state is located at the global energy minimum (Figure 4a), which is consistent with the experimental result [42,43] and our simulation results. Namely, the free DBD(CCG1) tends to stay in the unbound closed state during the simulations. It can also be seen from Figure 4a that there is also a local energy minimum around the half-open state. The local minimum indicates that the half-open state is metastable. There exists a considerably high energy barrier between the closed state and the CIA/ASF1-bound half-open state (Figure 4a), which is expected to be essential for DBD(CCG1) to recruit and transport CIA/ASF1. That is, the energy barrier will prevent DBD(CCG1) from transiting from the bound half-open state to the unbound closed state, even if CIA/ASF1 might get off DBD(CCG1) during the transportation of the DBD(CCG1)-CIA/ASF1 complex in cells, such that CIA/ASF1 would be able to easily bind back to DBD(CCG1).

Similar to the findings for unbound DBD(CCG1), there also exists local minima at the unbound closed and bound half-open states in the free energy profile of DBD(CCG1) in complex with CIA/ASF1 (Figure 4b). However, the two systems are thermodynamically very different. When DBD(CCG1) is free of CIA/ASF1, the unbound closed state is located at the global energy minimum and represents the most thermodynamically stable state in solution. Once DBD(CCG1) is in complex with CIA/ASF1, the binding of CIA/ASF1 will significantly favor the total free energy of the DBD(CCG1)-CIA/ASF1 complex and make the stable state of DBD(CCG1) transit from the closed state to the bound half-open state. As shown in the free energy landscape of CIA/ASF1-DBD(CCG1) system, the half-open state of DBD(CCG1) is located at the global energy minimum and thus more stable than the other states (Figure 4b), favoring the transportation of CIA/ASF1 with DBD(CCG1).

#### 2.2.2. The Moderate Energy Barrier between the Half-open State and the Open State Facilitates the Change of the Interacting Partner for CIA/ASF1

In addition to the closed state and the half-open state, there is also another metastable open conformational state in the free energy landscape of DBD(CCG1) (Figure 4a). However, unlike the high energy barrier between the closed and the half-open state, there is only a moderate free energy increase from the half-open state to the open state (Figure 4a). The moderate energy difference between the half-open state and the open state will facilitate the transference of CIA/ASF1 from DBD(CCG1) to histone H3-H4 because it makes the system easy to switch between the two states. As shown in our MD simulations, for DBD(CCG1) in complex with CIA/ASF1, the system mainly changes between the half-open state and the open state due to the much higher free energy of the closed state than the other states (Figure 4b). When the CIA/ASF1-bound DBD(CCG1) complex system changes to the open state from the half-open state, the hydrophobic interface between binding site 1a and CIA/ASF1 will be exposed to solvent, which will give histone H3-H4 a chance to interact with CIA/ASF1, resulting in the change of the interacting partner of CIA/ASF1 from DBD(CCG1) to histone H3-H4. From the thermodynamic perspective, this process is also facilitated by the moderate free energy barrier between the half-open and the open states because the system only involves a moderate free energy penalty during the transference of CIA/ASF1 from DBD(CCG1) to histone.

### 2.3. Asymmetric Binding of CIA/ASF1 Results in the Open State

The TLS (Translation, Libration, and Screw) tensor analysis based on the crystallographic refinement, reveals that domain 1 shows a higher mobility than domain 2 of DBD(CCG1) [42]. This is consistent with our PCA analysis of CIA/ASF1-bound DBD(CCG1), in which the open state is caused by the movement of domain 1 (Figure 3d). The crystal structure of CIA/ASF1-bound DBD(CCG1) also shows that the binding site 1b has a larger binding interface (447 Å${}^{2}$) than binding site 1a (390 Å${}^{2}$). The interface between binding site 1b and CIA/ASF1 also involves more hydrophobic interactions than that between binding site 1a and CIA/ASF1 (Table S1). In addition, the hydrogen bonds between binding site 1b and CIA/ASF1 further increase the interaction between binding site 1b and CIA/ASF1 (Table S1). All of these suggest that the higher mobility of domain 1 is caused by the weaker binding of CIA/ASF1 to binding site 1a than to binding site 1b, which enables the system to open and close by moving the domain 1 of DBD(CCG1).

**Figure 5:**
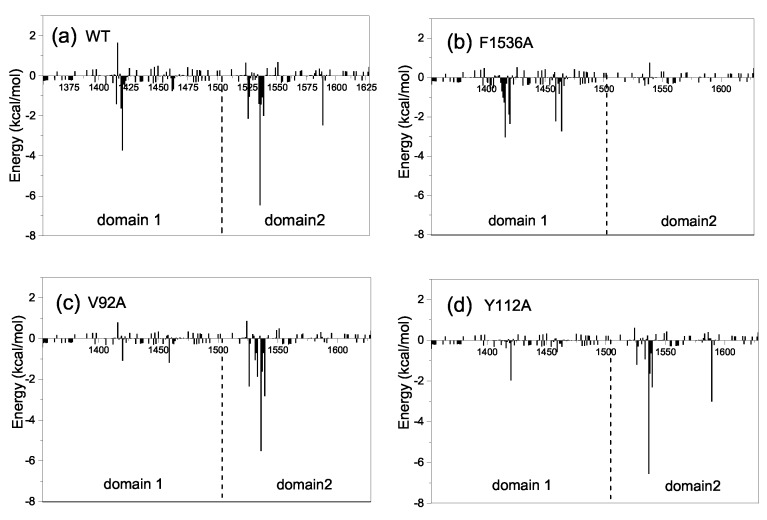
Decomposition of the binding free energy on per-residue basis for (**a**) wild type (WT), (**b**) F1536A, (**c**) V92A, and (**d**) Y112A. The left side of the dotted line shows the energy contributions of domain 1, and the right shows the energy contributions of domain 2. The detailed energy data of key residues are listed in Supplementary Table S2.

**Figure 6:**
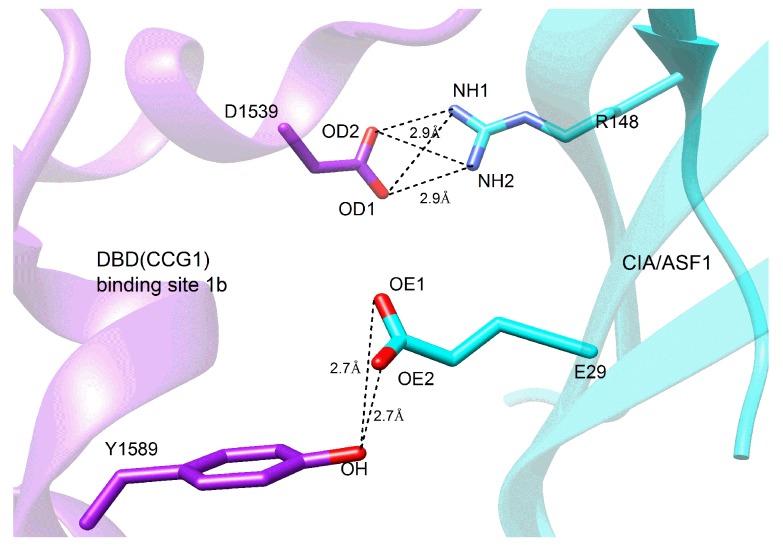
Important hydrogen bonds between DBD(CCG1) and CIA/ASF1.The hydrogen bonds are mainly formed between binding site 1b and CIA/ASF1. The structure is the average one from the last 20 ns of the MD trajectories of CIA/ASF1-bound DBD(CCG1).

To further investigate the molecular mechanism of the conformational change from the half-open to the open state, we also decomposed the binding free energy between CIA/ASF1 and DBD(CCG1) based on a short equilibrium in the last 20 ns of MD simulations for the half-open state of CIA/ASF1-bound DBD(CCG1). It can be found from the energy decomposition, the residues that have a large energy contribution (≤−1 kcal/mol) are mainly located at the ZA loop and BC loop of DBD(CCG1) and the hydrophobic regions of CIA/ASF1 (Figure 5a). These regions contribute over 90% to the binding free energy between CIA/ASF1 and DBD(CCG1), in which the energy contributions from binding site 1b (−31.27 kcal/mol) are 3.6 times more than that of binding site 1a (−8.69 kcal/mol) (Table S2). The hydrophobic interaction of the average structure is similar to that of the crystal structure, in which the interaction at binding site 1b is stronger than that at binding site 1a (Table S1). Moreover, the dynamic hydrogen bond network is mainly formed between binding site 1b and CIA/ASF1 (Figure 6 and Table S3). These results further indicate that the binding between binding site 1b and CIA/ASF1 is much stronger than that between binding site 1a and CIA/ASF1.

Based on the crystal structure and our MD simulations of the CIA/ASF-DBD(CCG1) system, it can be concluded that the transition from the half-open to the open state of CIA/ASF1-bound DBD(CCG1) is due to the asymmetric binding of CIA/ASF1 to DBD(CCG1). Namely, the much weaker binding of CIA/ASF1 at binding site 1a than binding site 1b enables one end of CIA/ASF1 to move away from domain 1 of DBD(CCG1) while the other end of CIA/ASF1 is still tightly attached to domain 2 of DBD(CCG1). As such, the free domain 1 of DBD(CCG1) would have an opportunity to recognize the acetylated *N*-terminal region of histone H4, and meanwhile the exposed surface of CIA/ASF1 has the chance to bind to histone H3-H4, resulting in the transference of CIA/ASF1 from DBD(CCG1) to histone.

### 2.4. Mechanism of the Effect of Key Residue Mutations on the Change of Binding State

The previous study revealed that the mutations of residues from binding site 1 (F1536A, Y1589A) and binding site 2 (H1610A, F1509A, Y1607A) of DBD(CCG1) and CIA/ASF1 (Y112A, V10A, V92A, V94A) play an important role in the binding of DBD(CCG1) to CIA/ASF1 [42]. In order to investigate the effects of these residue mutations on DBD(CCG1)-CIA/ASF1 binding, the nine mutant systems mentioned above were studied.

Among the nine mutant systems, the residue mutations of F1536A, V92A, and Y112A—which are located at the binding interface between CIA/ASF1 and DBD(CCG1)—directly affect the binding state change. Therefore, we mainly focus on the effects of F1536A, V92A, and Y112A on the binding state change. Based on the angles between domain 1 and domain 2 and RMSDs (Figures S1 and S2), it can be seen that the mutations V92A and Y112A make it easier for the system to transit among the three states. These two systems finally stay in the unbound closed state, while the wild type (WT) complex prefers to stay in the half-open state. The F1536A system does not show a binding state transition, although its RMSD has a large fluctuation (Figure S1). We also performed an ensemble cluster analysis for all the mutant systems and calculated their domain–domain angles and RMSDs (Figure S3). Similar to WT, the mutant systems also confirmed that the change of binding state is mainly caused by the movement of domain 1 and rotation of CIA/ASF1, while the binding site 1b tightly binds to CIA/ASF1 during the binding state change (Figure S4).

The interaction spectra of the mutant systems are similar to the WT system, except for F1536A, V92A, and Y112A (Figure 5 and Figure S5). The mutation of F1536A at binding site 1b, where DBD(CCG1) directly interacts with CIA/ASF1, results in a significantly conformational fluctuation of DBD(CCG1). The mutation of F1536A is unfavorable to the interaction between DBD(CCG1) and CIA/ASF1. Compared to the wild type, where the binding free energy between binding site 1b and CIA/ASF1 is −31.27 kcal/mol, the F1536A mutant led to a significant binding energy loss of −5.87 kcal/mol (Figure 5b, Tables S2 and S3). For the systems of V92A and Y112A, their binding states have a large change in the MD simulations and finally stay in the unbound closed state. Both electrostatic (ΔE${}_{ele}$) and van der Waals (ΔE${}_{vdw}$) energies have a significant decrease (Table S2). The interactions between binding site 1a and CIA/ASF1 of Y112A and V92A are almost nonexistent (Figure 5), which makes DBD(CCG1) easy to transit from the half-open state to the closed state and causes the complex to become unstable in the transportation process when disturbed by other factors in cells. The mutations of these residues, which have a significant effect on the CIA/ASF1-DBD(CCG1) complex, may mainly break the asymmetric interactions of binding site 1a and 1b of DBD(CCG1) with CIA/ASF1 and thus affect the transport process and partner change of CIA/ASF1 from DBD(CCG1) to histone H3-H4.

## 3. Discussion

**Figure 7:**
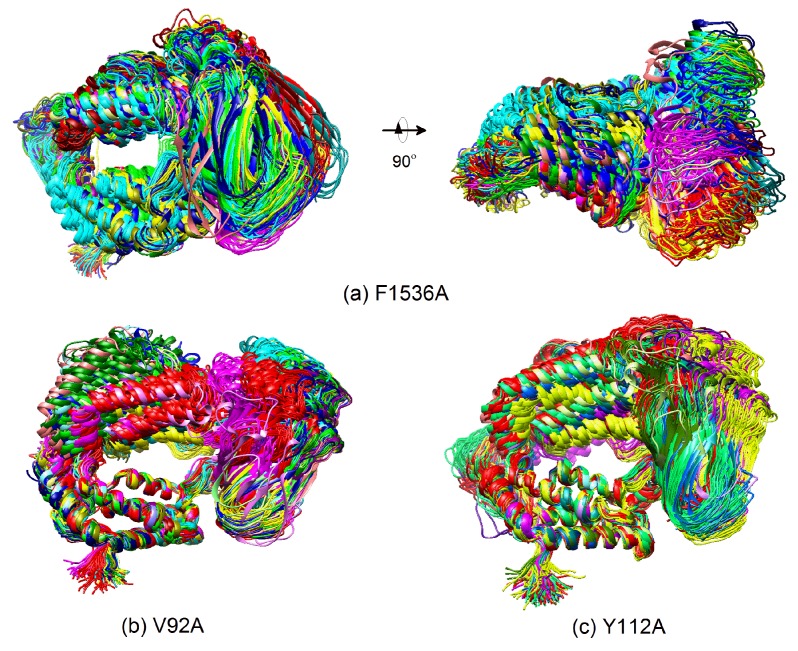
Ensemblecluster analysis of (**a**) F1536A, (**b**) V92A, and (**c**) Y112A. The mutation of F1536A does not lead to a binding state change, although its RMSD values show a large fluctuation. The mutations of V92A and Y112A make the conformation easier to change among the three states. Combining these with the angle analysis between two domains, it can be revealed that these two systems finally stay in the unbound closed binding state, while the wild type (WT) complex prefers to stay in the half-open state.

The structure-based biochemical and biological studies revealed that CIA/ASF1 colocalized with DBD(CCG1) at the promoter region through the interaction with DBD(CCG1) and is transferred to the histone H3-H4 [34,42]. However, the crystal structure of CIA/ASF1-DBD(CCG1) complex indicates that the hydrophobic interaction regions of CIA/ASF1 for histone H3-H4 and DBD(CCG1) are overlapped, which suggests an essential dynamic pathway of the interaction transference for CIA/ASF1 [36,42,43,46]. It was proposed that DBD(CCG1) recruits and transports CIA/ASF1 to nucleosome through binding site 1b within the half-open state; and CIA/ASF1 changes its interacting partner from DBD(CCG1) to histone H3-H4 in the open state. Through extensive MD simulations, we have revealed three thermodynamically metastable conformational states, the unbound closed state, the half-open state, and the open state. The conformational change from the half-open state to the open state enables the binding interface between binding site 1a of DBD(CCG1) and CIA/ASF1 to expose to solvent, which would give opportunities for binding site 1a of DBD(CCG1) to recognize the acetylated *N*-terminal tail of histone H4 and also for the corresponding region of CIA/ASF1 to bind to histone H3-H4 [42,50]. The crystal structure of CIA/ASF1-bound DBD(CCG1) shows that binding site 1b has a stronger interaction with CIA/ASF1 than binding site 1a [42]. The energy calculation indicates that the interaction energy for binding site 1b is 3.6 times more than that for binding site 1a. The weaker interaction at binding site 1a than 1b leads to a higher mobility for domain 1 of DBD(CCG1), which enables the systems to open and close by moving domain 1 of DBD(CCG1). When CIA/ASF1-DBD(CCG1) is in the open state, H3-H4 gets the chance to interact with CIA/ASF1. The CIA/ASF1-H3-H4 complex will be formed because of their strong interaction. Finally, binding site 1b of DBD(CCG1) would release the CIA/ASF1 to histone H3-H4, owing to the competitive binding of H3-H4 and DBD(CCG1) to CIA/ASF1. The proposed mechanism is also consistent with the experimental findings about the impact of residue mutations on the function of CIA/ASF1-DBD(CCG1). It has been reported in the literature that the mutation F1536A of DBD(CCG1) and mutations V92A and Y112A of CIA/ASF1 all adversely affected the biological process of the system. However, the mechanisms of these mutants are different according to the present dynamic pathway. For F1536A at binding site 1b of DBD(CCG1), its negative impact is mainly due to the weaker ability of DBD(CCG1) to recruit CIA/ASF1 because the mutant will significantly reduce the binding tightness between binding site 1b of DBD(CCG1) and CIA/ASF1. As shown in our MD simulations, this is indeed the case (Figure 7a and Figure 5b). Compared to the wild type where the binding free energy between binding site 1b and CIA/ASF1 is −31.27 kcal/mol, the F1536A mutant gave a significantly worse binding energy of −5.87 kcal/mol (Figure 5b and Table S2).

For the mutations of V92A and Y112A of CIA/ASF1, they are located at the binding site 1a and therefore have a different molecular mechanism. As found in our present study, DBD(CCG1) and CIA/ASF1 will separate from each other at binding site 1a to form an open conformational state due to their weak binding, where the corresponding binding interfaces will turn to recognize their interacting partners, i.e., the acetylated *N*-terminal region of histone H4 and the histone H3-H4 dimer, respectively. Therefore, the impact of these mutants are mostly due to their lower ability of DBD(CCG1) and CIA/ASF1 binding to their corresponding interacting partners because these mutations significantly reduce the hydrophobic surface around the regions and thus decrease their binding with other proteins. In addition, compared to the wild type, the mutations will also give DBD(CCG1) and CIA/ASF1 more flexibility to move apart due to their weaker binding at binding site 1a between each other (Figure 7b,c). As shown in our MD simulations, the interactions between DBD(CCG1) and CIA/ASF1 at binding site 1a for Y112A and V92A are almost disappeared (Figure 5 and Table S2), which makes DBD(CCG1) easy to transit from the bound half-open state to the unbound closed state and decrease its ability to recruit CIA/ASF1.

Molecular dynamics (MD) simulations have been a useful tool to study the biomolecular interactions at atom level, and may reveal more information than experimental methods can give [52,53,54]. However, the MD simulations is still computationally expensively. It is difficult to study the biological process that requires a long time, such as the complete process of CIA/ASF1 changes its interaction partner from CIA/ASF1 to H3-H4. In order to address this problem, a good choice would be to model the intermediates state by molecular modeling, then use the MD simulations to optimize the states, and finally combine all the intermediate states to form the whole biological process.

## 4. Materials and Methods

### 4.1. Protein Systems Preparation

The structure of CIA/ASF1-bound complex was obtained from the Protein Data Bank (PDB ID: 3AAD) [42]. The missing loop of CIA/ASF-bound structure was modeled by the MODELLER software [55]. The unbound DBD(CCG1) was obtained by removing CIA/ASF1 from the CIA/ASF1-bound structure. The mutants were generated by the PYMOL software [56]. The residue numbers of DBD(CCG1) (1353–1628) and CIA/ASF1 (1–153) were referenced from the crystal structure of 3AAD. The residues for mutation analysis were selected from binding site 1 (F1536A and Y1589A) and binding site 2 (F1509A, Y1607A, and H1610A) of DBD(CCG1) and CIA/ASF1 (V10A, V92A, V94A, and Y112A). These residues were reported to play a key role in the interaction between DBD(CCG1) and CIA/ASF1 in previous experiment studies [36,42,43]. We used the web-server H++ [57,58,59] to determine the protonation states and added hydrogen atoms for all protein structures. To ensure the integrity of systems, CIA/ASF1 at binding site 2 of DBD(CCG1) was retained during our simulations. As the interaction between CIA/ASF1 and binding site 1 of DBD(CCG1) was essential for their colocalization at active promoter sites, we mainly focused on the interactions between binding site 1 of DBD(CCG1) and CIA/ASF1 in this study.

### 4.2. Molecular Dynamics Simulation Protocol

The AMBER14 package [60] was used to perform MD simulations, where the AMBER ff14SB force field was adopted for protein structures, and the Leap module was used to generate the topology and coordinate files. All the protein structures were solvated in a truncated octahedron periodic water box of TIP3P [61] model with a cutoff of 10 Å. The Na${}^{+}$ or Cl${}^{-}$ counterions were used to neutralize the negative or positive charge of the systems. The systems were subjected to MD simulations with periodic boundary conditions. The cutoff value of no-bond interactions was set as 10 Å. The long-range electrostatic interactions were calculated by the Particle Mesh Ewald (PME) method [62]. The SHAKE algorithm [63] was used to constrain all the bonds involving hydrogen atoms.

All systems were subjected to a minimization and equilibration procedure. The minimization included three steps. First, the systems were subjected to 2500 steps of steep descent movements followed by 2500 steps of conjugate gradient minimization, to remove the bad clashes between solute and solvent. Second, the systems were gradually heated from 0 to 300 K in 50 ps. Finally, the systems were minimized at NVT ensemble for 50 ps. In the minimization procedure, the atoms of protein structures were restrained by a harmonic restraints of 2.0 kcal/(mol·Å${}^{2}$). Subsequently, the systems were equilibrated using Langevin dynamics under the constant-temperature and constant-pressure (NPT) conditions at 300 K and 1 atm for 250 ps without any position restraints. Then, the production simulations were performed at NPT (300 K, 1 atm) ensemble with a 2 fs time step. The conformational snapshots were saved for further analysis every 50 ps. The total MD simulations time for all systems was over 2.5 μs.

### 4.3. MD Trajectory Analysis

The analyses—including RMSD, principal component analysis (PCA), hydrogen bond, angle analysis, and distance between two residues—were done with the cpptraj module of AMBERTOOLS 14. Hydrogen bonds were counted with a distance cutoff of 3.5 Å between two heavy atoms and an angle cutoff of 120${}^{\circ }$ at intervening hydrogen atoms. The hydrogen bonds were characterized by the percentage of trajectory during which they are observed. The ensemble cluster analysis for the MD trajectory, interactional surface calculation, and trajectories visualization were done by the Chimera software [64]. The hydrophobic interaction and hydrogen bond analysis of the crystal structure and average structure were done by PIC [65].

### 4.4. Free Energy Calculations

As the protein conformational changes and interactions are determined by the free-energy landscape [52,53,54,66,67,68], we have constructed the free energy profile of the system. The MM-GBSA method [69,70] implemented in AMBER 14 was used to calculate the free energy of unbound DBD(CCG1) and CIA/ASF1-bound DBD(CCG1). The free energy was calculated by the follow equation:

(1) $$\Delta \Delta G_{TOT}=\Delta E_{MM}+\Delta G_{sol}-T\Delta S.$$

The binding free energy ΔΔG${}_{TOT}$ consists of the molecular mechanics free energy (ΔE${}_{MM}$), the solvation free energy (ΔG${}_{sol}$), and the conformational entropy effect on binding (−TΔS) in the gas phase. The ΔE${}_{MM}$ and ΔG${}_{sol}$ could be estimated by the following equations:

(2) $$\Delta E_{MM}=\Delta E_{ele}+\Delta E_{vdw},$$

(3) $$\Delta G_{sol}=\Delta G_{GB}+\Delta G_{SA}.$$

The ΔE${}_{MM}$ can be further divided into electrostatic interactions ΔE${}_{ele}$ and van der Waals energy ΔE${}_{vdw}$ in the gas phase, respectively. The solvation free energy (ΔG${}_{sol}$) can be divided into polar (ΔG${}_{GB}$) and nonpolar part (ΔG${}_{SA}$). The ΔG${}_{sol}$ was calculated with the GB module (IGB = 2) of the AMBER 14. In this paper, the dielectric constant was set to 1.0 for the interior solute and 80.0 for the exterior solvent. The same atomic radii and charges to MD simulations were used to calculate the binding free energy. The nonpolar contribution of the solvation free energy (ΔG${}_{SA}$) was determined according to the follow equation:

(4) $$\Delta G_{SA}=\gamma \times SASA+\beta ,$$

where the Solvent-Accessible Surface Area (SASA) was calculated by the MSMS algorithm, with a solvent probe radius of 1.4 Å. The empirical constants γ and β were set to 0.005 kcal/(mol·Å${}^{2}$) and 0.0, respectively. The entropy term (−TΔS) was estimated by a normal mode analysis with the NMODE module in the AMBER14. The decomposition of binding free energy was also done by the MM-GBSA module.

## 5. Conclusions

In the study, we have studied the dynamic mechanism of recruitment and transference of histone chaperone CIA/ASF1 through extensive MD simulations, where CIA/ASF1 is recruited to the promoter region by DBD(CCG1) and then changes its interacting partner from DBD(CCG1) to histone. It found that there exists three thermodynamically metastable conformational states—the unbound closed state, the CIA/ASF1-bound half-open state, and the open state—explaining the dynamic mechanism of how DBD(CCG1) stably recruits and transport CIA/ASF1 to the histone of nucleosome. It also showed that there is a high energy barrier between the closed and half-open bound states of DBD(CCG1) in the free energy landscape, which favors the stable binding of CIA/ASF1 to DBD(CCG1) during recruitment and transportation, as the energy barrier will prevent DBD(CCG1) from changing itself to the closed state, even if CIA/ASF1 may get on and off DBD(CCG1) during the transportation. The asymmetric binding of CIA/ASF1 to domains 1 and 2 of DBD(CCG1) leads to the presence of a crucial metastable open state where DBD(CCG1) and CIA/ASF1 will recognize their respective interacting partners, resulting in the transference of CIA/ASF1 from DBD(CCG1) to histone H3-H4. The moderate energy increase between the half-open state and the open state also facilitates the transference for CIA/ASF1 due to the small free energy penalty for the transference. The present model may present a general molecular mechanism for the recruitment and transference in protein–protein interactions.

## Supplementary Materials

The following are available online at https://www.mdpi.com/1422-0067/20/13/3325/s1, Figure S1: The RMSDs of all systems for C${}_{\alpha }$ atoms relative to the starting structure. The corresponding structures of unbound DBD(CCG1), WT, F1536A, V92A, and Y112A systems, which show large fluctuation of RMSDs, were extracted from the MD simulations. The structures extracted from MD simulations are colored in cornflower blue, and the crystal structure of unbound DBD are colored in orange. The structures of F1536A are in upward view. Figure S2: The angle curves between two domains of DBD(CCG1) for all mutant systems versus time. The mutations of V92A and Y112A have a great effect on the binding state, and their binding state finally located at the closed binding state. Figure S3: Ensemble cluster analysis of (a) Y1589A, (b) Y1607A, (c) F1509A, (d) H1610A, (e) V94A, and (f) V10A systems. These mutant systems do not show binding state change in the MD simulations. Figure S4: Principal component analysis of (a) F1536A, (b) V92A, (c) Y112A, (d) V10A, (e) Y1589A, (f) Y1607A, (g) F1509A, (h) H1610A, and (i) V94A systems. The binding state changes of V92A and Y112A are mainly caused by the movement of domain 1 and the rotation of CIA/ASF1. Figure S5: Decomposition of the binding free energy on the residue basis for (a) Y1589A, (b) H1610A, (c) F1509A, (d) Y1607A, (e) V94A, and (f) V10A systems. The left side of the dotted line shows the energy contribution of domain 1; and the right shows the energy contribution of domain 2. The interaction spectra of these systems are similar to the WT system with a few exceptions. Table S1: The hydrophobic interactions and hydrogen bond analysis of crystal structure and average structure of last 20 ns of CIA/ASF1-DBD(CCG1). Table S2: The binding energy contributions of key residues. Table S3: Hydrogen bonds between CIA/ASF1 and DBD for all systems at the binding site 1 in the last 20 ns.
