# Supplementary material for: Dynamics and Mechanisms in the Recruitment and Transference of Histone Chaperone CIA/ASF1
Source: Int J Mol Sci. 2019 Jul 6;20(13):3325. doi: 10.3390/ijms20133325 (PMC6651421; doi:10.3390/ijms20133325)
Supplement: Supplementary file 1 [file ijms-20-03325-s001.pdf]

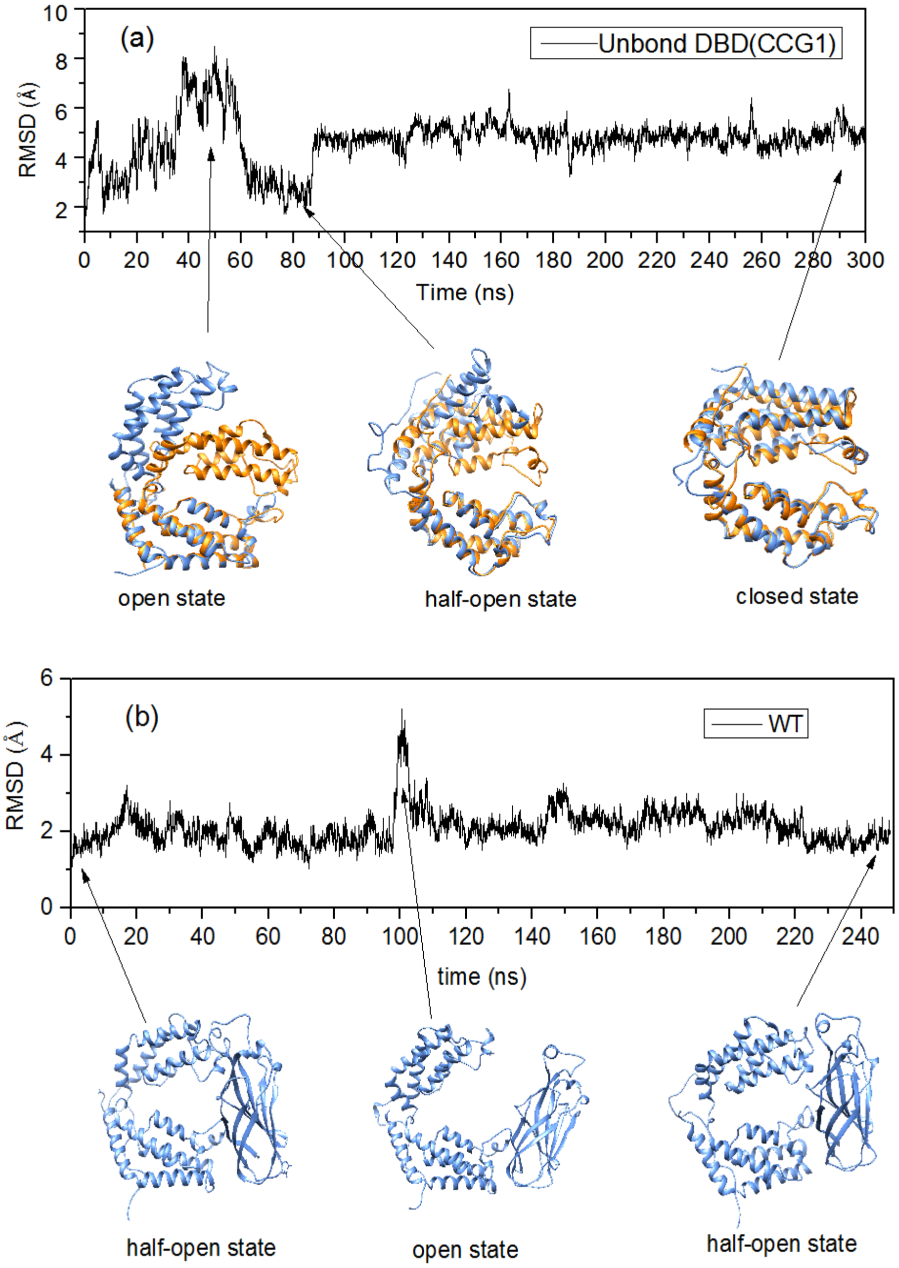

(a)
Unbond DBD(CCG1)
RMSD (Å)
Time (ns)
open state
half-open state
closed state
(b)
WT
RMSD (Å)
time (ns)
half-open state
open state
half-open state

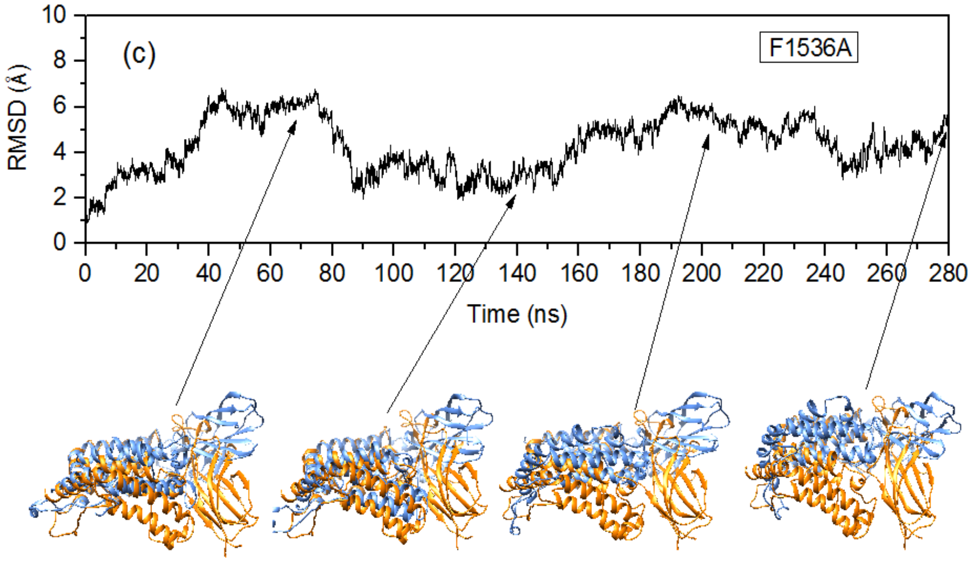
(c)
F1536A
RMSD (Å)
Time (ns)
0
2
4
6
8
10
0
20
40
60
80
100
120
140
160
180
200
220
240
260
280

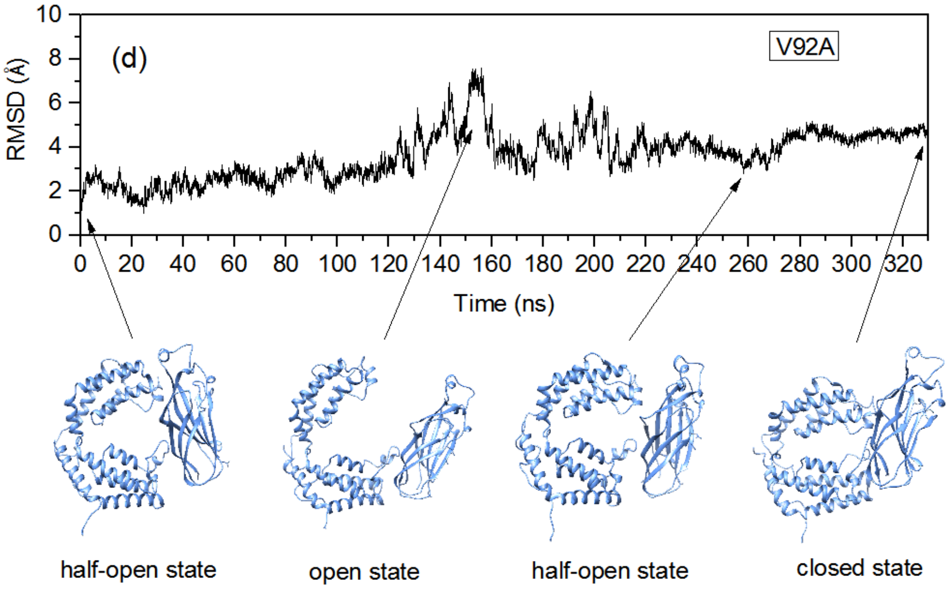
(d)
V92A
RMSD (Å)
Time (ns)
0
2
4
6
8
10
0
20
40
60
80
100
120
140
160
180
200
220
240
260
280
300
320
half-open state
open state
half-open state
closed state

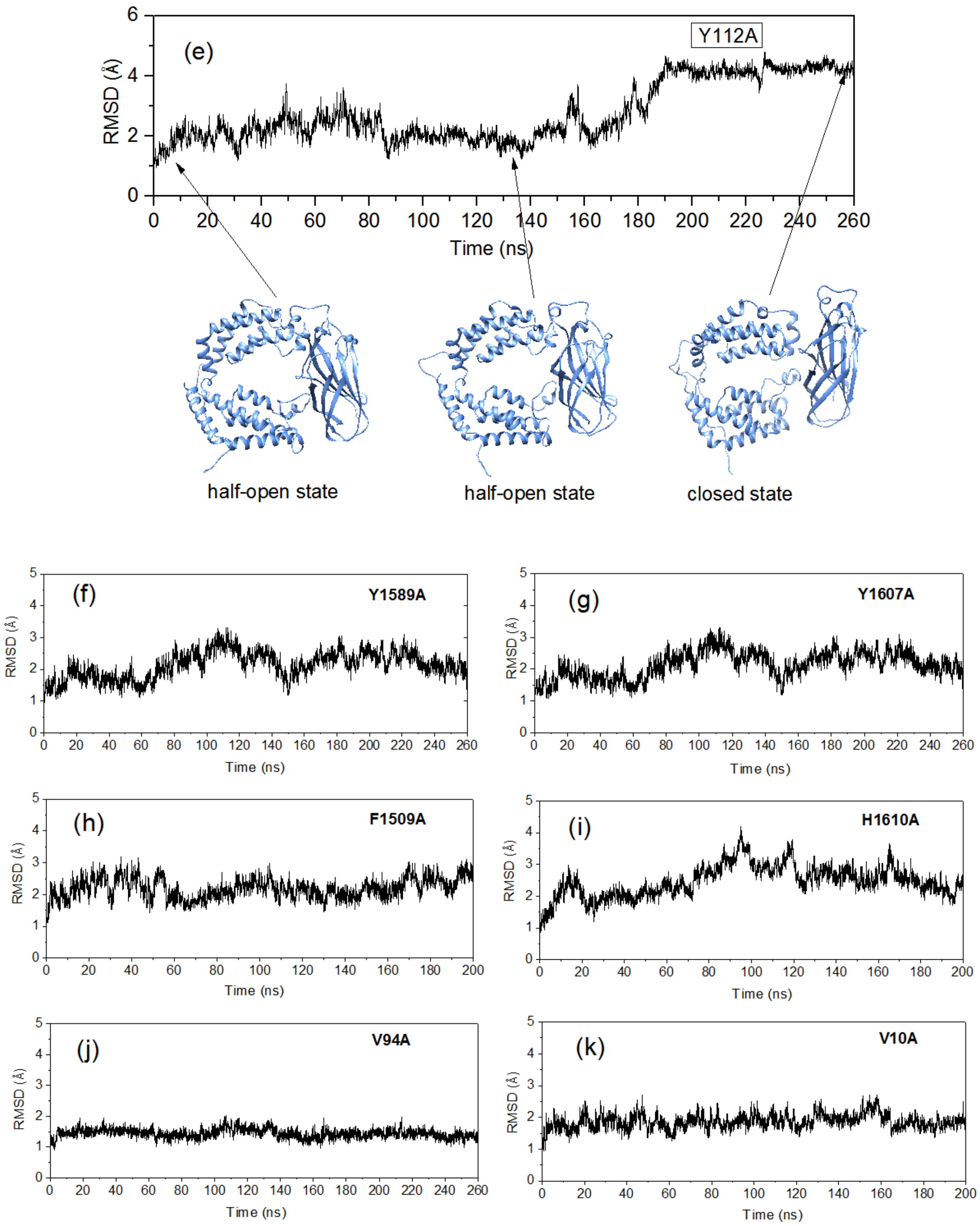

Figure S1. The RMSDs of all systems for Cα atoms relative to the starting structure. The corresponding structures of unbound DBD(CCG1), WT, F1536A, V92A, and Y112A systems, which show large fluctuation of RMSDs, were extracted from the MD simulations. The structures that were extracted from MD simulations were colored in cornflower blue, and the crystal structure of unbound DBD was colored in orange. The structures of F1536A were in upward view.

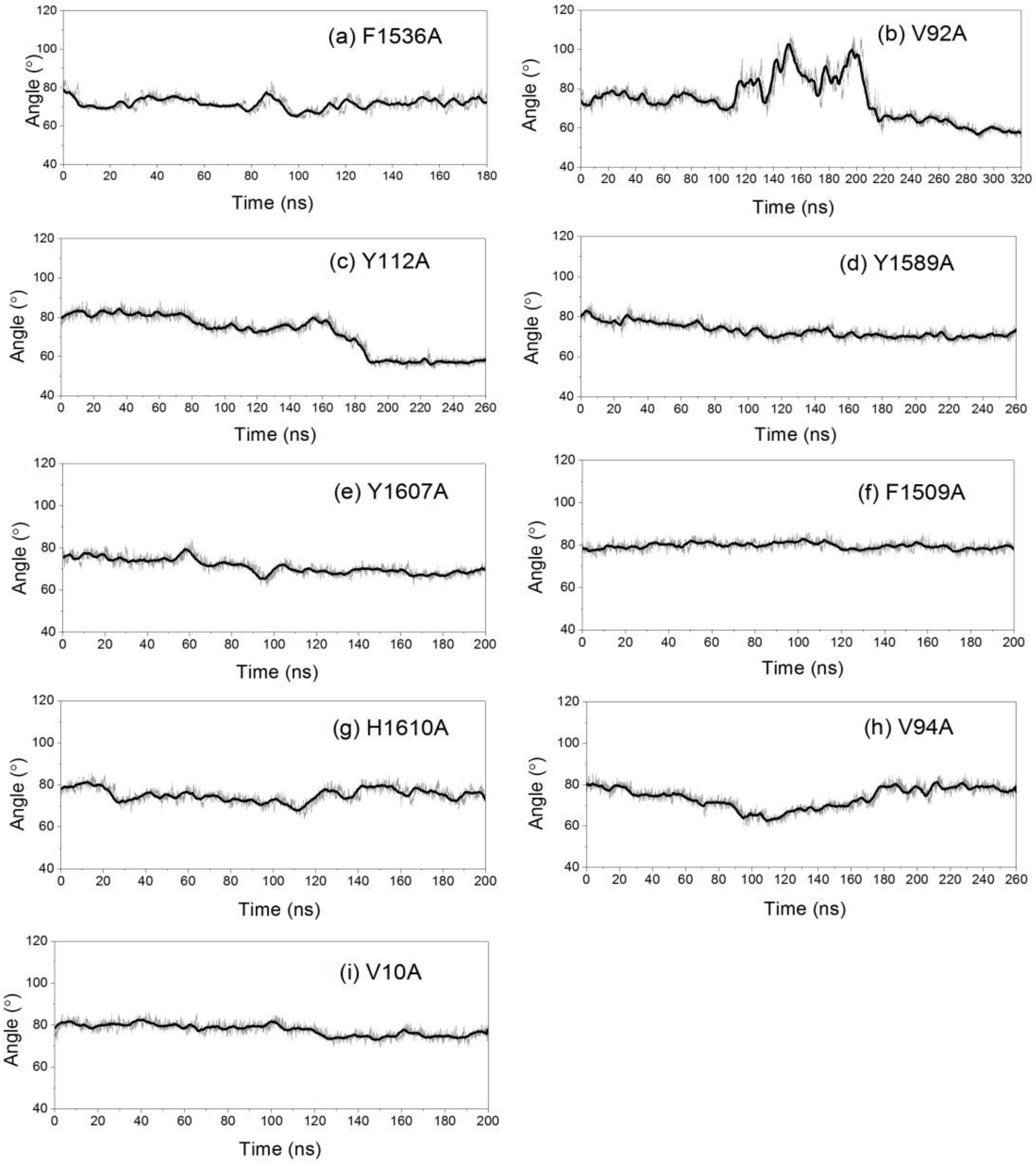

Figure S2. The angle curves between two domains of DBD(CCG1) for all mutant systems versus time. The mutations of V92A and Y112A have a great effect on the binding state, and their binding state finally located at the closed binding state.

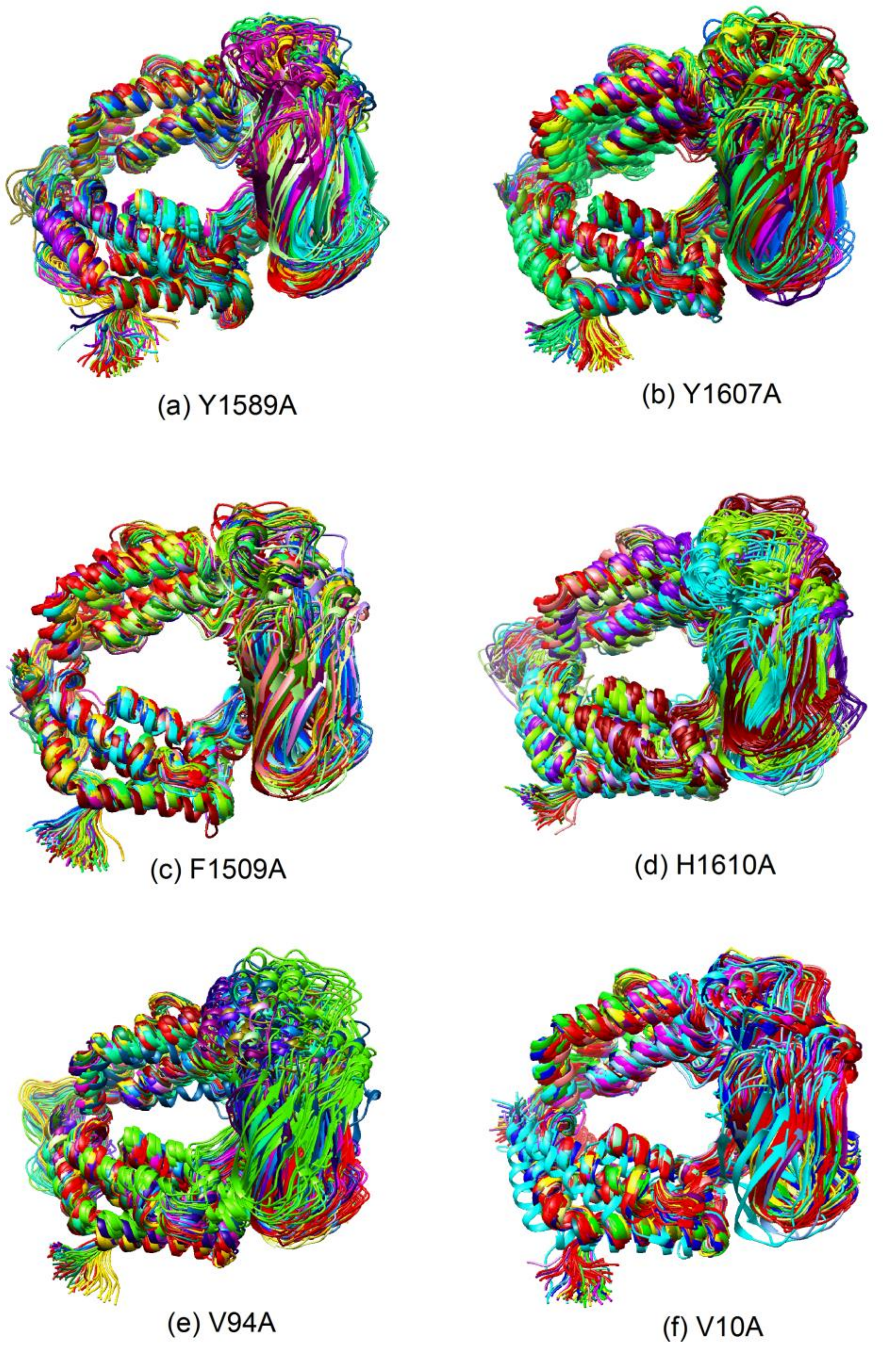

Figure S3. Ensemble cluster analysis of (a) Y1589A, (b) Y1607A, (c) F1509A, (d) H1610A, (e) V94A, and (f) V10A systems. These mutant systems do not show binding state change in the MD simulations.

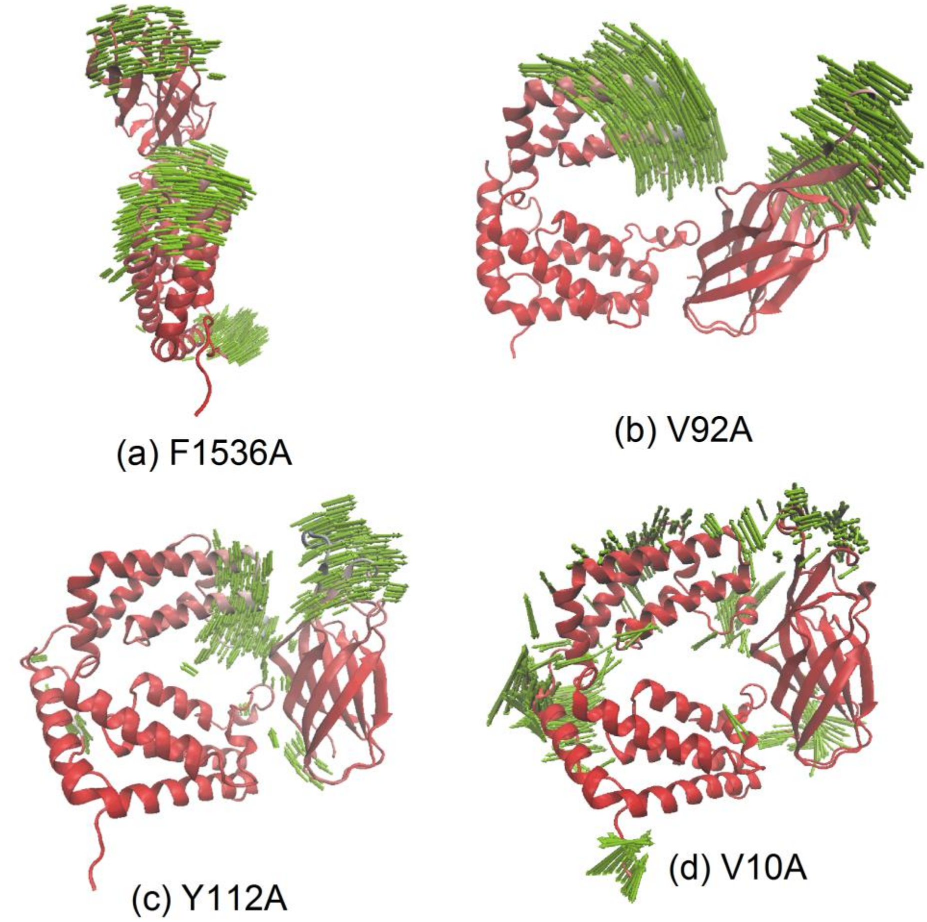
(a) F1536A

(b) V92A

(c) Y112A

(d) V10A

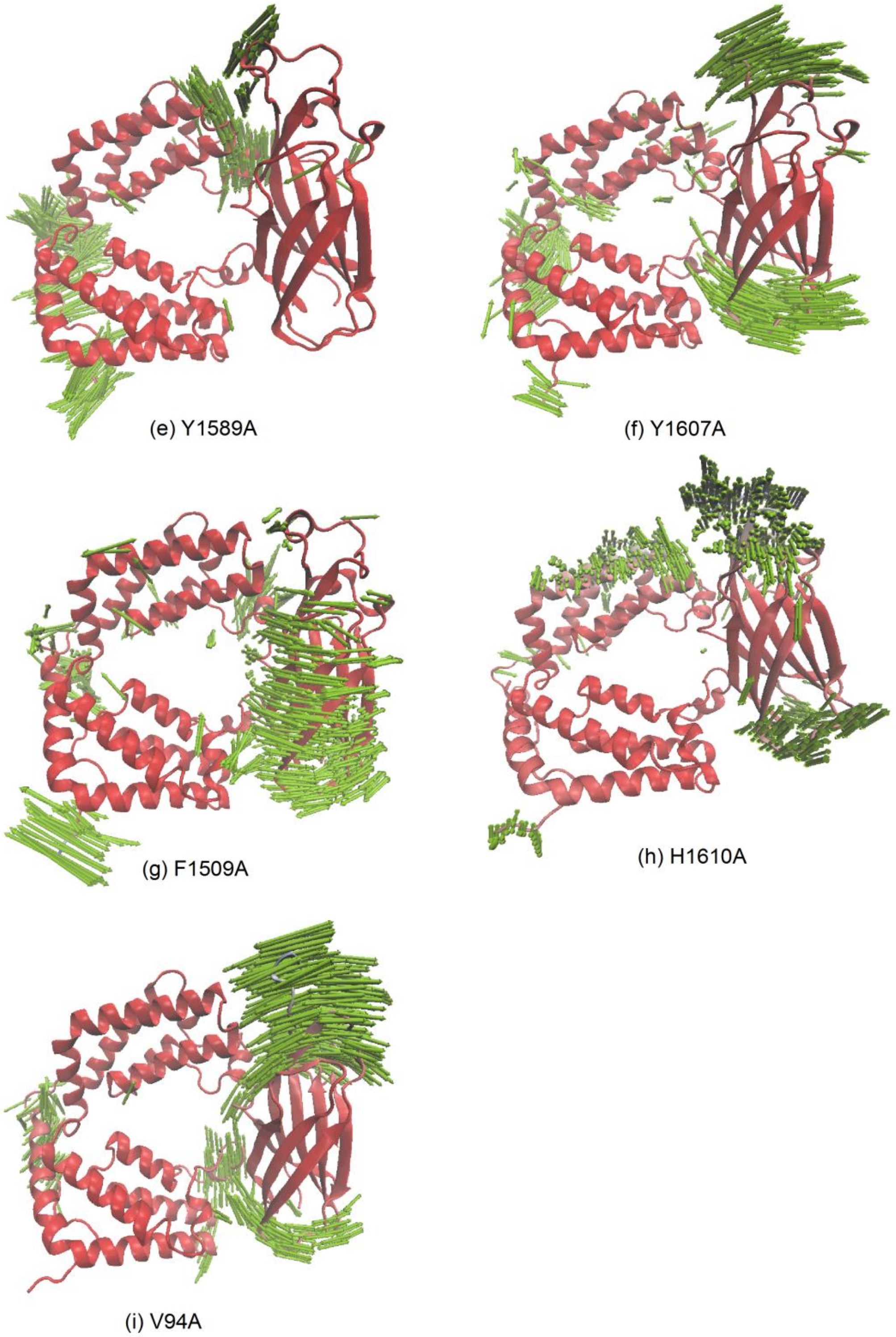

Figure S4. Principal component analysis of (a) F1536A, (b) V92A, (c) Y112A, (d) V10A, (e) Y1589A, (f) Y1607A, (g) F1509A, (h) H1610A, and V94A systems. The binding state changes of V92A and Y112A are mainly caused by the movement of domain 1 and the rotation of CIA/ASF1.

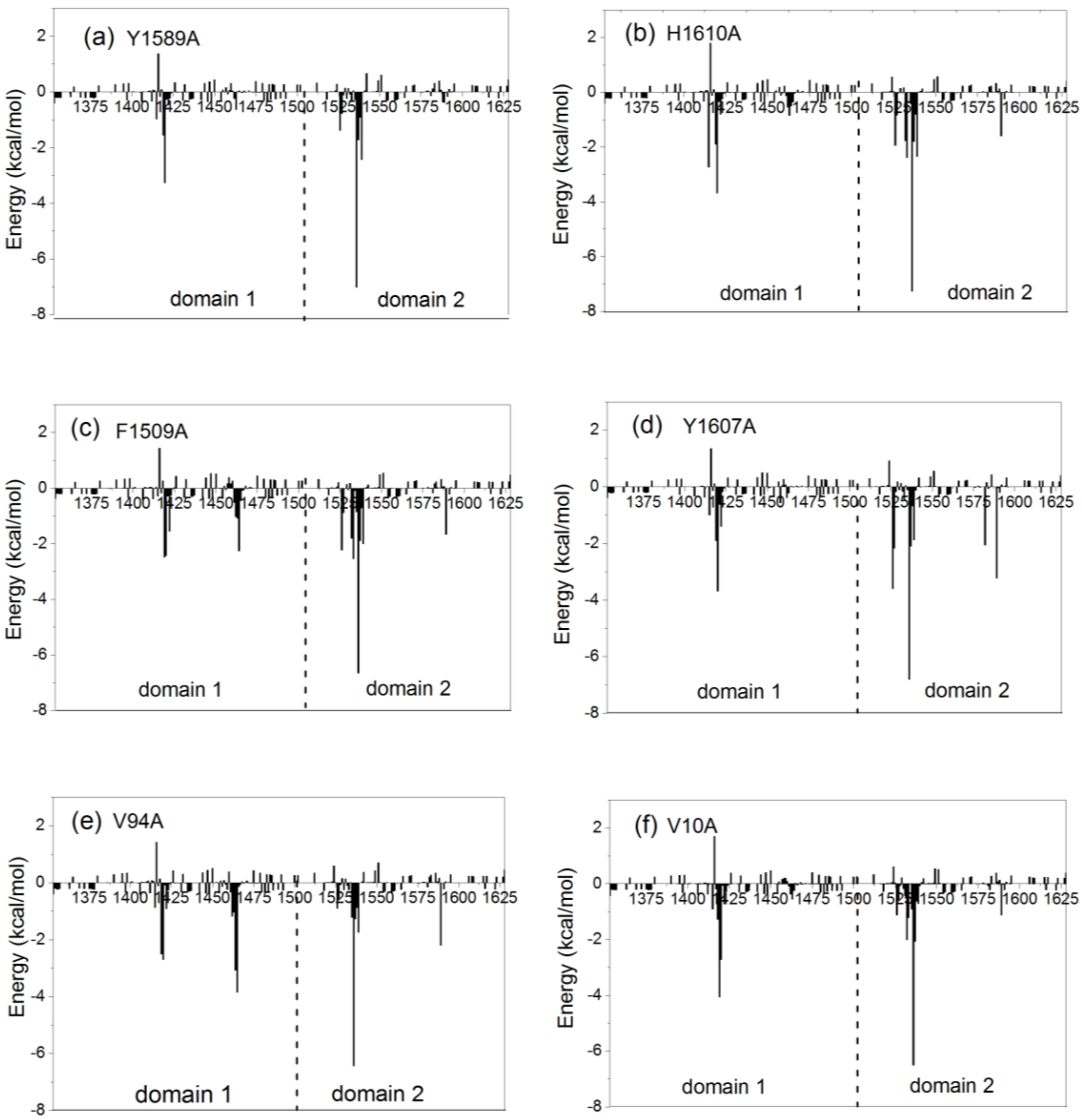

Figure S5. Decomposition of the binding free energy on the residue basis for (a) Y1589A, (b) H1610A, (c) F1509A, (d) Y1607A, (e) V94A, and (d) Y1607A systems. The left side of the dotted line shows the energy contribution of domain 1 and the right shows the energy contribution of domain 2. The interaction spectra of these systems are similar to the WT system with a few exceptions.

Table S1. The hydrophobic interactions and hydrogen bond analysis of crystal structure and average structure of last 20 ns of CIA/ASF1-DBD(CCG1).

**Crystal structure of CIA/ASF1-DBD(CCG1)**	
**Hydrophobic Interactions**	
**Binding site 1a**	
ILE1420-LEU140	ILE1420-VAL92
TYR1459-VAL92	TRP1526-VAL10
**Binding site 1b**	
PHE1536—ILE107	PHE1536-VAL109
PHE1536-VAL109	PHE1536-TYR111
PHE1536-PRO144	PHE1536-VAL146

PHE1536-VAL6	PHE1536-VAL9	
**Hydrogen bond**		
**Acceptor**	Donor	Distance (Å)
TRY1589	ARG69	3.42
**Average structure of last 20 ns trajectory**		
**Hydrophobic Interactions**		
Binding site 1a		
**ILE1420-LEU140**	TRP1526-MET71	
**Binding site 1b**		
PHE1536-ILE107	PHE1536-VAL109	
PHE1536-TYR111	PHE1536-PRO144	
PHE1536-VAL6	PHE1536-VAL9	
VAL1537-VAL6	PRO1538-VAL146	
**Hydrogen bond**		
**Acceptor**	Donor	Distance (Å)
ASN143@OD1	LYS1535@NZ	2.65
GLU29@OE1	TYR1589@OH	3.35
GLU29@OE2	TRY1589@OH	3.28
ASP1539@OD1	ARG148@NH1	2.92
ASP1539@OD1	ARG148@NH1	2.92
ASP1539@OD2	ARG148@NH1	2.86
ASP1539@OD2	ARG148@NH1	2.86
ASP1539@OD1	ARG148@NH2	2.91
ASP1539@OD1	ARG148@NH2	2.91
ASP1539@OD2	ARG148@NH2	2.83
ASP1539@OD2	ARG148@NH2	2.83

Table S2. The binding energy contributions of key residues for all systems. Vdw van der Waals energy; ele, electrostatic energy; GB, polar solvation energy; SA, non-polar solvation energy; S, side-chain atoms; B, back-bone atoms; T, total energy. All values are given in kcal/mol.

Residue	S_{VDW}	B_{VDW}	T_{VDW}	S_{ELE}	B_{ELE}	T_{ELE}	S_{GB}	B_{GB}	T_{GB}	T_{SUR}	T_{GBTOT}
**WT**											
**K1415**	-0.14	-0.07	-0.22	-50.58	-10.71	-61.29	49.39	10.8	60.19	-0.09	-1.41
**K1419**	-1.03	-0.74	-1.77	-48.11	-9.73	-57.84	47.43	10.91	58.34	-0.36	-1.63
**I1420**	-2.68	-0.68	-3.35	-1.41	-1.45	-2.87	1.34	1.62	2.96	-0.46	-3.72
**K1535**	-2.08	-1.05	-3.13	-37.85	-6.96	-44.81	38	9.03	47.03	-0.48	-1.39
**F1536**	-6.72	-1.29	-8	-1.9	-1.68	-3.58	2.81	3.19	6.01	-0.89	-6.46
**V1537**	-1.39	-0.39	-1.77	-0.52	1.61	1.09	0.49	-1.02	-0.54	-0.2	-1.43
**P1538**	-1.02	-0.22	-1.24	0.26	2.49	2.75	0.04	-2.46	-2.42	-0.17	-1.07
**D1539**	0.74	-0.17	0.58	-29.06	1.74	-27.32	26.62	-1.68	24.93	-0.18	-2
**Y1589**	-0.94	-0.03	-0.97	-6.33	0.57	-5.76	5.1	-0.54	4.55	-0.3	-2.47
**N8**	-1.27	-0.65	-1.92	-2.08	0.86	-1.21	3.42	-1.09	2.33	-0.14	-0.95
**V9**	-0.73	-0.69	-1.42	1.09	-3.02	-1.93	-1.12	3.43	2.31	-0.07	-1.11
**Y112**	-2.79	-0.13	-2.92	1.18	-1.93	-0.75	0.04	2.02	2.06	-0.32	-1.93
**L140**	-1.89	-0.24	-2.13	1.48	-2.78	-1.3	-1.31	3.24	1.93	-0.3	-1.8
**N143**	-2.21	-0.6	-2.81	-12.05	2.28	-9.77	11.89	-1.22	10.68	-0.46	-2.37
**P144**	-1.72	-0.34	-2.06	16.56	-26.38	-9.82	-16.18	25.02	8.84	-0.3	-3.34
**V146**	-2.08	-0.39	-2.46	1.23	-3.03	-1.8	-1.21	3.44	2.23	-0.3	-2.34
**R148**	-0.25	-0.04	-0.29	21.81	11.53	33.34	-25.95	-11.25	-37.2	-0.39	-4.54
**F1536A**											
**V1414**	-0.82	-0.4	-1.22	-0.9	-0.43	-1.34	0.92	0.72	1.64	-0.1	-1.01

**K1415**	-1.33	-0.4	-1.73	-49.3	-14.15	-63.45	49.6	14.51	64.11	-0.19	-1.26
**D1416**	-0.62	-0.85	-1.48	26.36	8.65	35.01	-28.22	-7.92	-36.14	-0.43	-3.04
**K1419**	-2.11	-0.37	-2.48	-65.73	-10.74	-76.47	66.07	11.4	77.47	-0.39	-1.88
**I1420**	-2.02	-0.22	-2.24	-1.17	0.7	-0.47	1.2	-0.53	0.67	-0.32	-2.36
**Y1459**	-2.62	-0.67	-3.29	-3.1	-1.18	-4.27	3.78	1.87	5.65	-0.31	-2.23
**H1464**	-0.74	-0.08	-0.81	-8.14	-2.98	-11.12	6.39	3.05	9.44	-0.24	-2.73
**S47**	0.82	-0.42	0.41	-11.03	-4.29	-15.33	4.44	2.9	7.34	-0.08	-7.66
**A48**	-1.46	-0.58	-2.04	2.33	-4.19	-1.86	-2.26	3.55	1.29	-0.21	-2.83
**E49**	-0.85	-0.19	-1.04	-63.37	-22	-85.37	63.31	20.05	83.37	-0.18	-3.24
**A87**	-0.89	-1.03	-1.92	2.76	-3.75	-0.99	-2.73	4.57	1.84	-0.31	-1.38
**D88**	-0.41	0	-0.41	-17.85	0	-17.85	13.5	0	13.5	-0.18	-4.93
**V92**	-2.45	-1.21	-3.66	1.32	0.25	1.57	-1.17	0.61	-0.56	-0.31	-2.95
**N114**	-1.55	-0.18	-1.73	-2.68	-0.62	-3.3	2.78	0.74	3.53	-0.22	-1.72
**L140**	-1.51	-0.11	-1.62	1.45	-1.66	-0.21	-1.37	1.87	0.5	-0.18	-1.5
**Y1589A**											
**K1415**	-0.11	-0.08	-0.2	-47.11	-10.33	-57.45	46.28	10.45	56.73	-0.07	-0.98
**K1419**	-1.76	-1.02	-2.78	-38.42	-7.95	-46.38	38.44	9.57	48.01	-0.41	-1.56
**I1420**	-2.52	-0.89	-3.41	-1.28	-0.91	-2.19	1.22	1.52	2.74	-0.41	-3.27
**K1535**	-2.27	-0.84	-3.11	-32.71	-7.55	-40.26	34.37	9.24	43.6	-0.51	-0.28
**F1536**	-6.86	-1.11	-7.97	-1.87	-2.88	-4.75	2.84	3.74	6.58	-0.89	-7.02
**V1537**	-1.66	-0.43	-2.09	-0.67	1.57	0.91	0.64	-0.96	-0.32	-0.22	-1.72
**P1538**	-0.92	-0.21	-1.12	-0.13	2.67	2.54	0.42	-2.63	-2.21	-0.13	-0.92
**D1539**	0.53	-0.17	0.36	-28.18	1.75	-26.43	25.54	-1.71	23.83	-0.2	-2.44
**N8**	-1.32	-0.69	-2.01	-2.87	0.97	-1.9	4.11	-1.2	2.9	-0.14	-1.14
**V9**	-0.7	-0.64	-1.34	1.13	-3.17	-2.04	-1.15	3.58	2.43	-0.07	-1.02
**Y112**	-2.81	-0.12	-2.92	-0.28	-2.05	-2.33	1.16	2.14	3.3	-0.36	-2.32
**L140**	-1.74	-0.17	-1.91	1.49	-2.57	-1.08	-1.35	2.95	1.6	-0.28	-1.68
**N143**	-2.55	-0.62	-3.18	-10	2.48	-7.52	10.05	-1.28	8.78	-0.46	-2.38
**P144**	-1.46	-0.2	-1.66	16.83	-26.55	-9.72	-16.42	24.66	8.24	-0.24	-3.38
**V146**	-2.19	-0.36	-2.55	1.45	-3.39	-1.94	-1.38	3.64	2.26	-0.3	-2.52
**R148**	-0.4	-0.05	-0.44	25.74	12.36	38.09	-30.11	-12.05	-42.16	-0.38	-4.88
**H1610A**											
**K1415**	0.03	-0.09	-0.06	-67.52	-10.71	-78.23	64.85	10.89	75.74	-0.18	-2.73
**K1419**	-1.69	-0.88	-2.57	-39.32	-9.53	-48.85	39.43	10.52	49.95	-0.41	-1.89
**I1420**	-2.66	-0.73	-3.39	-1.41	-2.05	-3.45	1.31	2.27	3.59	-0.43	-3.68
**W1526**	-2.57	-0.13	-2.7	-2.82	1.4	-1.41	3.64	-1.05	2.59	-0.42	-1.94
**V1532**	-1.38	-0.47	-1.85	-0.42	-1.24	-1.65	0.5	1.43	1.93	-0.19	-1.76
**N1533**	-1.4	-0.49	-1.89	-4.31	-1.75	-6.06	4.33	1.42	5.75	-0.2	-2.39
**F1536**	-7.38	-1.3	-8.69	-1.68	-3.6	-5.28	2.83	4.75	7.59	-0.89	-7.27
**V1537**	-1.86	-0.49	-2.36	-0.71	2.42	1.71	0.71	-1.64	-0.94	-0.21	-1.79
**D1539**	0.59	-0.15	0.44	-21.48	2.71	-18.78	18.65	-2.52	16.13	-0.15	-2.35
**Y1589**	-0.56	-0.02	-0.58	-5.28	0.22	-5.06	4.48	-0.2	4.28	-0.24	-1.59
**N7**	-1.73	-1.74	-3.47	1.25	-5.56	-4.31	0.15	6.82	6.98	-0.36	-1.16
**N8**	-1.76	-0.92	-2.68	-4.07	1.1	-2.97	4.91	-1.33	3.57	-0.23	-2.31
**V9**	-0.67	-0.64	-1.31	1.05	-3.12	-2.07	-1.07	3.54	2.46	-0.06	-0.97
**Y112**	-2.94	-0.13	-3.07	0.86	-1.93	-1.07	0.37	2.01	2.39	-0.32	-2.07
**L140**	-1.77	-0.18	-1.95	1.44	-2.37	-0.93	-1.27	2.74	1.47	-0.28	-1.69
**N143**	-1.7	-0.44	-2.14	-8.08	2.07	-6.01	7.87	-1.36	6.51	-0.34	-1.97
**P144**	-1.42	-0.48	-1.9	16.65	-20.74	-4.09	-16.21	20.43	4.22	-0.24	-2.01

**V146**	-2.25	-0.28	-2.53	1.26	-2.53	-1.26	-1.22	2.75	1.53	-0.27	-2.54
**R148**	-1.05	-0.06	-1.11	20.17	11.33	31.5	-24.49	-11.02	-35.51	-0.42	-5.53
**F1509A**											
**K1419**	-3	-1.52	-4.52	-46.8	-9.88	-56.69	47.29	12.05	59.34	-0.63	-2.49
**I1420**	-2.22	-0.93	-3.15	-1.54	1.09	-0.45	1.51	0.03	1.55	-0.38	-2.43
**T1422**	-0.93	-0.41	-1.34	-3.42	0.13	-3.29	3.15	0.27	3.42	-0.34	-1.55
**P1462**	-0.67	-0.22	-0.9	-10.06	8.93	-1.13	10.14	-9.04	1.1	-0.11	-1.03
**K1463**	-0.64	-0.48	-1.11	-45.37	-13.25	-58.62	45.15	13.66	58.81	-0.17	-1.09
**H1464**	-1.66	-0.33	-1.99	-4.61	-4.48	-9.09	4.44	4.7	9.14	-0.31	-2.26
**W1526**	-2.76	-0.17	-2.93	-2.27	1.82	-0.45	3.07	-1.46	1.61	-0.47	-2.24
**V1532**	-1.41	-0.48	-1.89	-0.44	-1.21	-1.64	0.52	1.4	1.92	-0.19	-1.8
**N1533**	-1.42	-0.52	-1.94	-4.35	-1.87	-6.22	4.32	1.48	5.8	-0.19	-2.55
**F1536**	-6.77	-1.14	-7.91	-1.74	-3.43	-5.17	2.77	4.51	7.29	-0.86	-6.65
**V1537**	-1.91	-0.44	-2.36	-0.74	2.5	1.76	0.74	-1.83	-1.09	-0.22	-1.9
**D1539**	0.78	-0.13	0.66	-20.02	3.04	-16.98	17.34	-2.88	14.46	-0.15	-2
**Y1589**	-0.48	-0.02	-0.5	-5.79	0.11	-5.67	4.84	-0.09	4.75	-0.23	-1.66
**N7**	-1.78	-1.79	-3.57	0.81	-5.61	-4.8	0.59	6.79	7.38	-0.36	-1.36
**N8**	-1.87	-0.95	-2.82	-4.49	1.19	-3.29	5.36	-1.41	3.96	-0.24	-2.39
**V9**	-0.71	-0.75	-1.46	1.02	-3.37	-2.35	-1.05	3.85	2.8	-0.07	-1.08
**L140**	-3.01	-0.23	-3.24	1.46	-2.78	-1.32	-1.33	3.04	1.71	-0.38	-3.23
**S142**	-0.21	-0.25	-0.46	0.85	-7.52	-6.67	-1.18	7.53	6.35	-0.21	-0.98
**P144**	-1.16	-0.3	-1.47	15.92	-20.97	-5.06	-15.54	20.39	4.85	-0.2	-1.87
**V146**	-1.81	-0.23	-2.04	1.21	-2.51	-1.3	-1.18	2.71	1.54	-0.26	-2.07
**R148**	-0.57	-0.05	-0.62	19.54	10.77	30.31	-23.65	-10.49	-34.15	-0.39	-4.84
**Y1607A**											
**K1415**	0.01	-0.06	-0.04	-42.34	-8.71	-51.06	41.36	8.79	50.14	-0.04	-1
**K1419**	-1.41	-0.75	-2.16	-38.36	-9.53	-47.89	38.29	10.25	48.53	-0.39	-1.91
**I1420**	-2.56	-0.72	-3.28	-1.35	-2.23	-3.58	1.28	2.33	3.61	-0.43	-3.69
**T1422**	-1.38	-0.63	-2	-1.68	-0.62	-2.3	2.17	0.96	3.12	-0.22	-1.4
**F1536**	-6.87	-1.09	-7.96	-1.75	-2.62	-4.37	2.68	3.71	6.39	-0.87	-6.8
**V1537**	-2.08	-0.4	-2.48	-0.39	1.55	1.16	0.43	-0.95	-0.53	-0.25	-2.1
**D1539**	0.38	-0.18	0.2	-29.41	0.73	-28.68	27.35	-0.52	26.82	-0.23	-1.88
**Y1582**	-1.69	-0.09	-1.79	-2.01	-5.19	-7.19	2.44	4.76	7.21	-0.27	-2.05
**Y1589**	-2.83	-0.08	-2.91	-4.19	0.67	-3.51	4.28	-0.62	3.66	-0.46	-3.22
**N8**	-1.25	-0.69	-1.94	-2.65	0.81	-1.84	3.73	-1.1	2.63	-0.14	-1.3
**I31**	-1.44	-0.31	-1.75	1.09	-1.57	-0.48	-1.06	1.81	0.74	-0.24	-1.73
**R69**	-4.7	-0.66	-5.37	39.47	9.52	48.99	-36.43	-8.59	-45.02	-0.59	-1.99
**Y112**	-2.9	-0.12	-3.03	0.79	-1.76	-0.97	0.35	1.84	2.19	-0.29	-2.09
**L140**	-1.8	-0.25	-2.05	1.29	-2.43	-1.14	-1.15	2.87	1.72	-0.28	-1.74
**N143**	-2.38	-0.62	-3.01	-11.92	2.33	-9.6	10.78	-1.46	9.31	-0.36	-3.65
**P144**	-1.49	-0.47	-1.96	15.95	-22.14	-6.19	-15.56	21.44	5.89	-0.25	-2.52
**V146**	-1.93	-0.29	-2.23	1.28	-2.99	-1.71	-1.24	3.28	2.04	-0.27	-2.17
**R148**	-0.28	-0.04	-0.32	19.9	11.36	31.26	-24.67	-11.06	-35.73	-0.36	-5.15
**V94A**											
**K1419**	-2.65	-1.09	-3.74	-57.46	-10.91	-68.37	57.79	12.44	70.23	-0.62	-2.5
**I1420**	-2.72	-0.76	-3.48	-1.43	0.12	-1.3	1.4	1.09	2.49	-0.42	-2.7
**T1422**	-1.17	-0.23	-1.4	-1.88	0.16	-1.72	2.35	0.21	2.55	-0.34	-0.91
**P1462**	-0.8	-0.25	-1.05	-10.19	8.9	-1.29	10.36	-9.08	1.28	-0.11	-1.18
**K1463**	-0.84	-0.45	-1.29	-47.79	-12.86	-60.65	47.65	13.5	61.15	-0.24	-1.03

**H1464**	-2.17	-0.23	-2.4	-0.83	-8.04	-8.87	1.8	6.66	8.46	-0.26	-3.07
**S1465**	0.34	-0.2	0.14	-9.2	-3.54	-12.74	6.37	2.48	8.85	-0.09	-3.84
**K1535**	-2.03	-0.79	-2.82	-32.59	-7.12	-39.71	33.08	8.73	41.81	-0.49	-1.22
**F1536**	-6.7	-1.12	-7.82	-1.72	-1.83	-3.55	2.77	3.04	5.81	-0.87	-6.43
**V1537**	-1.28	-0.33	-1.6	-0.5	1.58	1.08	0.49	-1.04	-0.55	-0.19	-1.27
**P1538**	-0.86	-0.19	-1.05	0.31	2.3	2.61	-0.06	-2.24	-2.3	-0.13	-0.87
**D1539**	0.53	-0.15	0.38	-25.07	1.67	-23.4	23.06	-1.63	21.44	-0.16	-1.74
**Y1589**	-1.37	-0.03	-1.4	-5.51	0.68	-4.82	4.98	-0.66	4.33	-0.3	-2.2
**N8**	-1.33	-0.69	-2.02	-2.84	1.05	-1.79	3.99	-1.24	2.75	-0.15	-1.21
**V9**	-0.72	-0.67	-1.39	1.1	-3.02	-1.92	-1.12	3.45	2.33	-0.07	-1.05
**V92**	-1.86	-0.26	-2.11	1.44	-0.92	0.53	-1.36	1.2	-0.16	-0.32	-2.07
**Y112**	-2.43	-0.14	-2.57	1.19	-1.61	-0.42	-0.52	1.77	1.24	-0.22	-1.97
**L140**	-2.09	-0.19	-2.29	1.34	-2.66	-1.32	-1.34	2.98	1.64	-0.29	-2.25
**P144**	-1.45	-0.22	-1.67	16.63	-24.64	-8.01	-16.25	23.65	7.4	-0.27	-2.55
**V146**	-1.96	-0.3	-2.27	1.32	-2.84	-1.52	-1.26	3.08	1.82	-0.28	-2.24
**R148**	-0.66	-0.05	-0.71	21.76	11.33	33.08	-25.22	-11.03	-36.25	-0.4	-4.28
**V10A**											
**K1415**	-0.21	-0.12	-0.34	-46.07	-10.43	-56.5	45.33	10.65	55.98	-0.06	-0.91
**Y1418**	-0.46	-0.14	-0.6	-0.95	-3.83	-4.78	1.09	3.14	4.23	-0.13	-1.27
**K1419**	-3.12	-1.69	-4.81	-65.86	-8.99	-74.85	65.01	11.34	76.35	-0.75	-4.06
**I1420**	-2.24	-0.91	-3.15	-1.41	-0.51	-1.92	1.4	1.35	2.75	-0.39	-2.72
**W1526**	-1.96	-0.07	-2.03	-1.67	0.37	-1.3	2.75	-0.17	2.58	-0.37	-1.12
**V1532**	-1.44	-0.52	-1.97	-0.08	-0.98	-1.05	0.1	1.07	1.17	-0.17	-2.02
**N1533**	-1.72	-0.46	-2.18	-2.23	-2.67	-4.89	3.68	2.39	6.07	-0.2	-1.22
**F1536**	-6.81	-1.13	-7.94	-1.37	-3.63	-5	2.7	4.61	7.3	-0.88	-6.51
**V1537**	-2.01	-0.41	-2.41	-0.48	1.74	1.26	0.49	-1.18	-0.69	-0.23	-2.07
**Y1589**	-1.93	-0.03	-1.96	-2.16	0.91	-1.25	3.27	-0.87	2.4	-0.33	-1.13
**N8**	-2.04	-0.67	-2.71	-1.56	1.71	0.15	2.73	-1.13	1.6	-0.32	-1.28
**A48**	-0.37	-0.04	-0.41	2.55	-7.73	-5.17	-2.52	7.2	4.68	-0.07	-0.97
**E49**	-0.09	-0.12	-0.22	-84.14	-12.78	-96.93	83.54	12.44	95.97	-0.17	-1.34
**V92**	-1.27	-0.13	-1.4	1.46	-0.77	0.69	-1.39	0.96	-0.43	-0.2	-1.33
**V109**	-1.24	-0.22	-1.46	1.05	0.81	1.86	-1.08	-0.92	-2	-0.1	-1.7
**Y112**	-2.56	-0.13	-2.69	1.47	-1.62	-0.15	-0.72	1.69	0.97	-0.17	-2.04
**L140**	-1.75	-0.17	-1.92	1.2	-2	-0.81	-1.15	2.39	1.24	-0.23	-1.72
**S142**	-1.87	-0.94	-2.81	-0.27	-6.08	-6.35	0.51	6.75	7.25	-0.45	-2.36
**N143**	-1.1	-0.61	-1.71	-7.72	1.15	-6.56	7.11	-0.19	6.92	-0.23	-1.58
**P144**	-1.8	-0.55	-2.34	16.94	-24.19	-7.25	-16.46	23.03	6.57	-0.31	-3.33
**V146**	-1.74	-0.27	-2	1.38	-3.6	-2.22	-1.31	3.76	2.45	-0.26	-2.04
**R148**	-0.65	-0.03	-0.68	26.39	11.13	37.52	-27.94	-10.88	-38.81	-0.25	-2.22
**V92A**											
**I1420**	-1.04	-0.07	-1.1	-0.87	0.53	-0.35	0.85	-0.38	0.47	-0.1	-1.08
**F1536**	-5.98	-1.12	-7.1	-1.4	-3.26	-4.66	2.24	4.81	7.05	-0.8	-5.52
**V1537**	-1.77	-0.46	-2.23	-0.36	2.6	2.24	0.34	-1.74	-1.4	-0.23	-1.62
**D1539**	1.13	-0.12	1	-29.41	2.37	-27.04	25.51	-2.17	23.34	-0.13	-2.82
**V6**	-1.18	-0.97	-2.15	1.15	-2.78	-1.62	-1.12	4.08	2.96	-0.2	-1.01
**N7**	-1.17	-0.83	-2	2.88	-6.84	-3.96	-1.84	7.05	5.21	-0.25	-1
**I31**	-1.2	-0.4	-1.6	1.34	-1.36	-0.02	-1.25	1.78	0.53	-0.23	-1.32
**N143**	-2.85	-0.44	-3.29	-9.37	1.68	-7.7	10.57	-0.77	9.8	-0.59	-1.78
**V146**	-1.45	-0.16	-1.61	1.26	-2.52	-1.26	-1.22	2.82	1.59	-0.22	-1.49

**R148**	-0.76	-0.05	-0.8	26.38	12.01	38.39	-30.67	-11.7	-42.36	-0.46	-5.24
**Y112A**											
**I1420**	-1.74	-0.22	-1.96	-0.9	0.89	-0.01	0.88	-0.67	0.2	-0.19	-1.96
**W1526**	-1.8	-0.09	-1.89	-0.72	-0.48	-1.2	1.57	0.62	2.19	-0.29	-1.19
**F1536**	-6.55	-1.05	-7.6	-2.02	-2.01	-4.03	2.64	3.25	5.89	-0.83	-6.56
**V1537**	-1.58	-0.36	-1.94	-0.45	1.66	1.21	0.43	-1.12	-0.69	-0.21	-1.63
**D1539**	0.86	-0.13	0.73	-28.24	1.93	-26.3	25.29	-1.81	23.47	-0.2	-2.3
**Y1589**	-0.87	-0.03	-0.9	-7.21	0.37	-6.84	5.37	-0.35	5.02	-0.29	-3.01
**N8**	-1.48	-0.72	-2.2	-3.46	1	-2.46	4.8	-1.35	3.45	-0.18	-1.39
**V9**	-0.78	-0.79	-1.56	1.08	-3.96	-2.88	-1.12	4.48	3.37	-0.08	-1.15
**S142**	-1.59	-1.19	-2.77	1.35	-7.93	-6.58	-0.16	8.98	8.81	-0.42	-0.96
**N143**	-2.1	-0.7	-2.8	-7.87	1.47	-6.39	8.12	-0.88	7.23	-0.34	-2.29
**P144**	-2.2	-0.63	-2.82	16.51	-20.6	-4.09	-16	20.28	4.28	-0.31	-2.95
**V146**	-1.3	-0.21	-1.51	1.23	-3.54	-2.31	-1.17	3.84	2.67	-0.24	-1.4
**R148**	0.33	-0.03	0.3	22.8	11.86	34.66	-28.18	-11.58	-39.77	-0.31	-5.12

Table S3. Hydrogen bonds between CIA/ASF1 and DBD for all systems at the binding site 1 in the last 20 ns. For every system, the cut-off used for hydrogen bond distance and angle is 3.5 Å and 120°, respectively. The percent hydrogen bond occupancy that hydrogen bonds formed during the MD simulations is produced by the CPPTRAJ module of AMBER14. In addition, each hydrogen bond distance is measured between the heavy atom of donor and the heavy atom of acceptor.

#Acceptor	DonorH	Donor	Occupancy	Distance (Å)
**WT**				
**ASP_1539@OD1**	ARG_148@HH12	ARG_148@NH1	0.3797	2.9068
**ASP_1539@OD2**	ARG_148@HH22	ARG_148@NH2	0.3651	2.8988
**ASP_1539@OD2**	ARG_148@HH12	ARG_148@NH1	0.3404	2.9145
**GLU_29@OE2**	TYR_1589@HH	TYR_1589@OH	0.3376	2.7138
**ASP_1539@OD1**	ARG_148@HH22	ARG_148@NH2	0.3374	2.9175
**PHE_1536@HZ**	VAL_9@HB	VAL_9@CB	0.3223	3.3096
**GLU_29@OE1**	TYR_1589@HH	TYR_1589@OH	0.2596	2.7162
**ASP_1539@CG**	ARG_148@HH12	ARG_148@NH1	0.257	3.3652
**GLU_29@CD**	TYR_1589@HH	TYR_1589@OH	0.2254	3.2955
**ILE_1420@O**	ASN_143@HD21	ASN_143@ND2	0.221	2.9546
**ASP_1539@CG**	ARG_148@HH22	ARG_148@NH2	0.2047	3.384
**F1536A**				
**ASP_1416@OD2**	SER_47@HG	SER_47@OG	0.7855	2.6271
**ASP_1416@OD1**	ASH_88@HD2	ASH_88@OD2	0.7204	2.6179
**ASP_1416@OD2**	ALA_48@H	ALA_48@N	0.6173	3.0352
**ASP_1416@CG**	ASH_88@HD2	ASH_88@OD2	0.5943	3.1942
**TYR_1459@O**	ASN_114@HD22	ASN_114@ND2	0.5896	2.9069
**GLU_116@OE2**	HIE_1464@HE2	HIE_1464@NE2	0.4979	2.9558
**GLU_116@OE1**	HIE_1464@HE2	HIE_1464@NE2	0.4879	2.9605
**GLU_49@H**	LYS_1419@HD3	LYS_1419@CD	0.3823	3.2495
**GLU_116@CD**	HIE_1464@HE2	HIE_1464@NE2	0.3039	3.3548
**ASP_1416@HB2**	VAL_92@H	VAL_92@N	0.2273	3.1527
**ASN_114@HD22**	ASN_1458@HA	ASN_1458@CA	0.2152	3.3152
**Y1589A**				
**LYS_1535@O**	ARG_148@HH22	ARG_148@NH2	0.4833	2.8629
**ILE_1420@O**	ASN_143@HD21	ASN_143@ND2	0.3635	2.9587
**PHE_1536@HZ**	VAL_9@HB	VAL_9@CB	0.3304	3.3111
**ASP_1539@OD1**	ARG_148@HH22	ARG_148@NH2	0.2138	2.911
**ASP_1539@OD2**	ARG_148@HH12	ARG_148@NH1	0.2085	2.8811
**ASP_1539@OD2**	ARG_148@HH22	ARG_148@NH2	0.1881	2.9168
**ASP_1539@OD1**	ARG_148@HH12	ARG_148@NH1	0.1821	2.9134
**H1610A**				
**ASN_7@O**	ASN_1533@H	ASN_1533@N	0.5701	3.0806
**ASP_1539@OD2**	ARG_148@HH12	ARG_148@NH1	0.5279	2.8989
**ASP_1539@OD2**	ARG_148@HH22	ARG_148@NH2	0.5199	2.9152
**ASP_1539@OD1**	ARG_148@HH22	ARG_148@NH2	0.5166	2.894
**ASP_1539@OD1**	ARG_148@HH12	ARG_148@NH1	0.5074	2.899
**PHE_1536@HZ**	VAL_9@HB	VAL_9@CB	0.4396	3.2814
**ILE_1420@O**	ASN_143@HD21	ASN_143@ND2	0.3966	2.9578
**ASN_8@OD1**	ASN_1533@HD21	ASN_1533@ND2	0.3916	2.9372
**ASP_1539@CG**	ARG_148@HH12	ARG_148@NH1	0.3428	3.38
**ASP_1539@CG**	ARG_148@HH22	ARG_148@NH2	0.3253	3.387

**F1509A**				
**ASN_7@O**	ASN_1533@H	ASN_1533@N	0.6995	3.0705
**ASN_8@OD1**	ASN_1533@HD21	ASN_1533@ND2	0.4933	2.9258
**ASP_1539@OD2**	ARG_148@HH22	ARG_148@NH2	0.4547	2.8855
**ASP_1539@OD1**	ARG_148@HH12	ARG_148@NH1	0.4492	2.9022
**ASP_1539@OD2**	ARG_148@HH12	ARG_148@NH1	0.446	2.9018
**ASP_1539@OD1**	ARG_148@HH22	ARG_148@NH2	0.4405	2.8953
**THR_1422@OG1**	SER_142@HG	SER_142@OG	0.4368	2.7813
**PHE_1536@HZ**	VAL_9@HB	VAL_9@CB	0.4002	3.2966
**ASN_114@HB2**	ILE_1420@HG12	ILE_1420@CG1	0.3028	3.299
**ASP_1539@CG**	ARG_148@HH12	ARG_148@NH1	0.2965	3.3794
**THR_1422@HG1**	SER_142@HG	SER_142@OG	0.2883	3.3008
**ASP_1539@CG**	ARG_148@HH22	ARG_148@NH2	0.2567	3.388
**ILE_1420@HG12**	ASN_114@HB2	ASN_114@CB	0.2097	3.2636
**GLU_29@OE2**	TYR_1589@HH	TYR_1589@OH	0.2077	2.7471
**GLU_29@CD**	TYR_1589@HH	TYR_1589@OH	0.205	3.2722
**Y1607A**				
**ASP_1539@OD1**	ARG_148@HH12	ARG_148@NH1	0.5185	2.8836
**ASP_1539@OD2**	ARG_148@HH22	ARG_148@NH2	0.5095	2.8983
**ASP_1539@OD1**	ARG_148@HH22	ARG_148@NH2	0.5072	2.9039
**ASP_1539@OD2**	ARG_148@HH12	ARG_148@NH1	0.484	2.8955
**ILE_1420@O**	ASN_143@HD21	ASN_143@ND2	0.4522	2.9127
**ASP_1539@CG**	ARG_148@HH12	ARG_148@NH1	0.3857	3.3607
**ASP_1539@CG**	ARG_148@HH22	ARG_148@NH2	0.3553	3.3772
**GLU_29@OE2**	TYR_1589@HH	TYR_1589@OH	0.3445	2.7111
**PHE_1536@HZ**	VAL_9@HB	VAL_9@CB	0.3407	3.3045
**GLU_29@OE1**	TYR_1589@HH	TYR_1589@OH	0.2742	2.708
**TYR_1582@OH**	GLN_5@HE22	GLN_5@NE2	0.267	3.1446
**GLU_29@CD**	TYR_1589@HH	TYR_1589@OH	0.2482	3.3292
**Y112A**				
**ASN_7@O**	ASN_1533@H	ASN_1533@N	0.9067	3.0208
**PRO_1531@O**	ASN_8@HD21	ASN_8@ND2	0.8063	2.9184
**VAL_9@H**	PHE_1536@HE1	PHE_1536@CE1	0.246	3.2893
**TYR_1418@O**	SER_142@HG	SER_142@OG	0.2172	2.7232
**V10A**				
**ASN_7@O**	ASN_1533@H	ASN_1533@N	0.9067	3.0208
**PRO_1531@O**	ASN_8@HD21	ASN_8@ND2	0.8063	2.9184
**VAL_9@H**	PHE_1536@HE1	PHE_1536@CE1	0.246	3.2893
**TYR_1418@O**	SER_142@HG	SER_142@OG	0.2172	2.7232
**LEU_140@HD13**	ILE_1420@HA	ILE_1420@CA	0.1915	3.2379
**LEU_140@HD12**	ILE_1420@HA	ILE_1420@CA	0.1905	3.2397
**V92A**				
**ASP_1539@OD1**	ARG_148@HH22	ARG_148@NH2	0.5697	2.8826
**ASP_1539@OD2**	ARG_148@HH12	ARG_148@NH1	0.5685	2.8838
**ASP_1539@OD1**	ARG_148@HH12	ARG_148@NH1	0.5282	2.8937
**ASP_1539@OD2**	ARG_148@HH22	ARG_148@NH2	0.5157	2.8908
**ASN_7@O**	ASN_1533@HD21	ASN_1533@ND2	0.511	2.899
**ASP_1539@CG**	ARG_148@HH12	ARG_148@NH1	0.447	3.361
**ASP_1539@CG**	ARG_148@HH22	ARG_148@NH2	0.3832	3.3746

**ILE_31@O**	TYR_1589@HH	TYR_1589@OH	0.2774	2.8145
**PHE_1536@HB3**	VAL_6@HB	VAL_6@CB	0.2237	3.3259
**V94A**				
**ASP_1539@OD2**	ARG_148@HH12	ARG_148@NH1	0.4406	2.8998
**ASP_1539@OD1**	ARG_148@HH22	ARG_148@NH2	0.4358	2.9061
**ASP_1539@OD2**	ARG_148@HH22	ARG_148@NH2	0.4173	2.9093
**ASP_1539@OD1**	ARG_148@HH12	ARG_148@NH1	0.4104	2.9008
**PHE_1536@HZ**	VAL_9@HB	VAL_9@CB	0.3787	3.3133
**ASP_1539@CG**	ARG_148@HH12	ARG_148@NH1	0.3133	3.3763
**ASP_1539@CG**	ARG_148@HH22	ARG_148@NH2	0.2708	3.3817
**ILE_1420@O**	ASN_143@HD21	ASN_143@ND2	0.246	2.9383
